# Argonaute protein CSR-1 restricts localization of holocentromere protein HCP-3, the *C. elegans* CENP-A homolog

**DOI:** 10.1242/jcs.261895

**Published:** 2024-09-18

**Authors:** Charmaine Yan Yu Wong, Hok Ning Tsui, Yue Wang, Karen Wing Yee Yuen

**Affiliations:** ^1^ School of Biological Sciences, The University of Hong Kong, Hong Kong; ^2^ School of Biological Sciences, University of Southampton, Southampton SO17 1BJ, UK

**Keywords:** Centromere, Kinetochore, Argonaute, RNA interference, CENP-A, HCP-3, Chromosome segregation

## Abstract

Chromosome segregation errors caused by centromere malfunction can lead to chromosome instability and aneuploidy. In *Caenorhabditis elegans*, the Argonaute protein CSR-1 is essential for proper chromosome segregation, although the specific mechanisms are not fully understood. Here, we investigated how CSR-1 regulates centromere and kinetochore function in *C. elegans* embryos. We found that depletion of CSR-1 results in defects in mitotic progression and chromosome positioning relative to the spindle pole. Knockdown of CSR-1 does not affect mRNA and protein levels of the centromeric histone H3 variant and CENP-A homolog HCP-3 but does increase the localization of HCP-3 and some kinetochore proteins to the mitotic chromosomes. Such elevation of HCP-3 chromatin localization depends on EGO-1, which is an upstream factor in the CSR-1 RNA interference (RNAi) pathway, and PIWI domain activity of CSR-1. Our results suggest that CSR-1 restricts the level of HCP-3 at the holocentromeres, prevents erroneous kinetochore assembly and thereby promotes accurate chromosome segregation. Our work sheds light on the role of CSR-1 in regulating deposition of HCP-3 on chromatin and centromere function in embryos.

## Teaser

**Summary:** Argonaute CSR-1, in an RNA interference pathway, acts to restrict localization of the holocentromere protein HCP-3 and kinetochore proteins at the poleward faces of mitotic chromosomes in *C. elegans*.

## INTRODUCTION

Accurate chromosome segregation during cell division is crucial for organism survival and is moderated by the microtubules, which attach to chromosomes through the kinetochores. The kinetochore complex is assembled on the centromere region of each chromosome (Fukagawa and Earnshaw, 2014; McKinley and Cheeseman, 2016). When the nuclear envelope breaks down, kinetochore proteins assembled on the poleward sides of sister chromatids are captured by the microtubules. The condensed chromosomes congress to form the metaphase plate under different pulling and pushing forces exerting on the chromosomes (Tanaka, 2002; Levesque and Compton, 2001). After that, the sister chromatids segregate equally to the daughter cells. Erroneous microtubule attachment is prone to result in uneven chromosome segregation. One type of improper attachment is known as merotelic attachment, where a single kinetochore is captured by microtubules emanating from both spindle poles (Kapoor, 2004). This creates an imbalance in the pulling forces exerted on the chromosome, leading to lagging chromosomes or chromosome bridges between the segregating masses (Gregan et al., 2011). Proper bi-orientation and balanced tension across the kinetochore–microtubule attachments are crucial for accurate chromosome segregation during cell division.

Despite centromeres having a conserved function, centromere architecture varies among organisms. In *Caenorhabditis elegans*, the holocentromere is positioned along the entire poleward faces of the condensed sister chromatids (Buchwitz et al., 1999). The number of microtubules attached per chromosome is similar between human centromeres and *C. elegans* holocentromeres (McEwen et al., 1998; Redemann et al., 2017). In many organisms, the histone H3 variant CENP-A is a common marker of active centromeres, including ectopic centromeres and neocentromeres (Amor and Choo, 2002; De Rop et al., 2012). The CENP-A homolog in *C. elegans*, HCP-3, is found to be associated with ∼25–50% of the genome, which includes both genic and intergenic DNA regions (Gassmann et al., 2012; Lin et al., 2021). HCP-3 is at the top of the hierarchy for kinetochore assembly in *C. elegans*, and depletion of HCP-3 leads to kinetochore-null (knl) phenotypes and severe chromosome missegregation (Oegema et al., 2001). CENP-A in humans loads onto the centromeric genomic region via the specific chaperone HJURP through recognition of the Mis18 complex, which acts as a licensing factor (Foltz et al., 2009); however, HJURP is not found in *C. elegans.* To our current knowledge, the centromere licensing factor KNL-2, which is a Mis18BP1 homolog, and the histone chaperone LIN-53, which is a homolog of RbAp46 (also known as RBBP7) and RbAp48 (also known as RBBP4), are the only two known factors required for HCP-3 localization in *C. elegans* (Maddox et al., 2007; Lee et al., 2016). It is not clear whether other chromatin-modifying or epigenetic pathways have a role in regulating HCP-3 localization or deposition.

The Argonaute proteins are conserved effector proteins in the RNA interference (RNAi) pathway, which uses small RNAs to regulate complementary target transcripts. *C. elegans* has an extensive RNAi network driven by a large set of 27 Argonaute proteins (Yigit et al., 2006; Billi et al., 2018). Among these, the Argonaute protein chromosome segregation and RNAi-deficient-1 (CSR-1) plays a unique and essential role in ensuring proper chromosome segregation during embryonic cell divisions. Embryos depleted of CSR-1 exhibit high levels of genome instability and embryonic lethality, whereas homozygous *csr-1* null mutants are nearly sterile (Claycomb et al., 2009). The small interfering RNAs (siRNAs) bound by CSR-1 (22G siRNAs) are synthesized by the RNA-dependent RNA polymerase EGO-1, the Dicer-related helicase DRH-3 and the Tudor domain-containing protein EKL-1 (Claycomb et al., 2009). These 22G siRNAs are then uridylated, which regulates their binding to the Argonaute protein CSR-1 (van Wolfswinkel et al., 2009).

The CSR-1-bound 22G siRNAs are antisense to more than 4000 endogenous germline-expressed genes (Claycomb et al., 2009; Gu et al., 2009). CSR-1 has a catalytically active ‘DDH’ PIWI domain that has been proposed to cleave mRNA targets (Aoki et al., 2007). Whole-genome microarray expression analysis has revealed a complex effect after CSR-1 depletion, with some genes being upregulated and some being downregulated (Claycomb et al., 2009). The degree of suppression of germline mRNA gene expression by CSR-1 correlates with the corresponding siRNA abundance (Gerson-Gurwitz et al., 2016). In addition, CSR-1–siRNA complexes have been found to interact with the genomic position of their target, suggesting a potential chromatin function in addition to silencing (Wedeles et al., 2013b). CSR-1 has been implicated in mRNA maturation, translational regulation (Avgousti et al., 2012; Friend et al., 2012; Gerson-Gurwitz et al., 2016; Quarato et al., 2021) and protection of targets against PIWI-interacting RNA (piRNA)-induced silencing by antagonizing other germline RNAi pathways, such as those involving PRG-1 and HRDE-1 (Wedeles et al., 2013b; Seth et al., 2013).

However, the precise mechanisms by which CSR-1-mediated regulation of germline gene expression controls embryonic chromosome segregation have remained unclear. In CSR-1-depleted embryos, chromosomes fail to align tightly at metaphase, and lagging chromosomes and chromosome bridges are observed at anaphase (Yigit et al., 2006; Claycomb et al., 2009). These chromosome segregation defects could potentially stem from disruptions to the cytoskeleton that affect microtubules, the kinetochore–microtubule interface, or centromere structure and function. Notably, several lines of study suggest that CSR-1 might influence the inner centromere protein occupancy. A previous study has reported that the genomic regions targeted by CSR-1 tend to be depleted of the centromere histone variant HCP-3, suggesting a potential negative correlation (Gassmann et al., 2012). Another investigation has found that in *csr-1* hypomorphic embryos, HCP-3 occupancy is reduced in two flanking regions of CSR-1 targets, implying that CSR-1 might in fact have a positive effect on HCP-3 occupancy in flanking regions of CSR-1 targets (Cecere et al., 2014). These conflicting reports have left open questions on whether and how CSR-1 regulates the chromatin localization of centromere and kinetochore proteins. If CSR-1 does affect these proteins, understanding how CSR-1 regulates them and contributes to the observed chromosome segregation defects remains a challenge.

To address these questions, we conducted a comprehensive analysis of the effects of CSR-1 depletion. We assessed the functionality of kinetochore–microtubule attachment in the embryonic divisions in the absence of CSR-1. We quantified an increase in centromere and kinetochore protein localization on chromatin after CSR-1 depletion. We evaluated the HCP-3 expression upon CSR-1 depletion at the transcript and protein levels. Interestingly, although the total HCP-3 protein level was unchanged, more HCP-3 was deposited onto the chromatin. We further screened for requirements important for CSR-1 to control the HCP-3 level on chromatin. We found that upstream components of the CSR-1 RNAi pathway and the PIWI domain activity of CSR-1 were essential for this elevation of chromatin-localized HCP-3. Our work unravels details of the mechanism by which CSR-1 is required to regulate the proper amount of centromere protein deposited on holocentromeres, which has significant implications for our understanding of holocentromere function and organization.

## RESULTS

### Knockdown of CSR-1 leads to mitotic delays and changes in net force between spindle pole and chromatin

As CSR-1 is important for chromosome segregation, *csr-1* hypomorphic mutants are almost sterile, and embryos with *csr-1* depletion exhibit aneuploidy (Claycomb et al., 2009). Introduction of *csr-1* double-stranded RNA (dsRNA) to worms at the fourth larval stage (L4) by injection knocked down *csr-1* mRNA to 16% of its original level (Fig. S1A). We found that 80% of the F1 progeny of the injected worms had an embryonic lethal phenotype (Fig. S1B). In embryos with RNAi-mediated knockdown of CSR-1 [hereafter referred to as *csr-1*(RNAi)], we observed lagging chromosomes in 70% of the anaphase cells (Fig. S1C), and 30% of *csr-1*(RNAi) embryonic cells were classified as aneuploid (Fig. S1D).

**Fig. 1.**
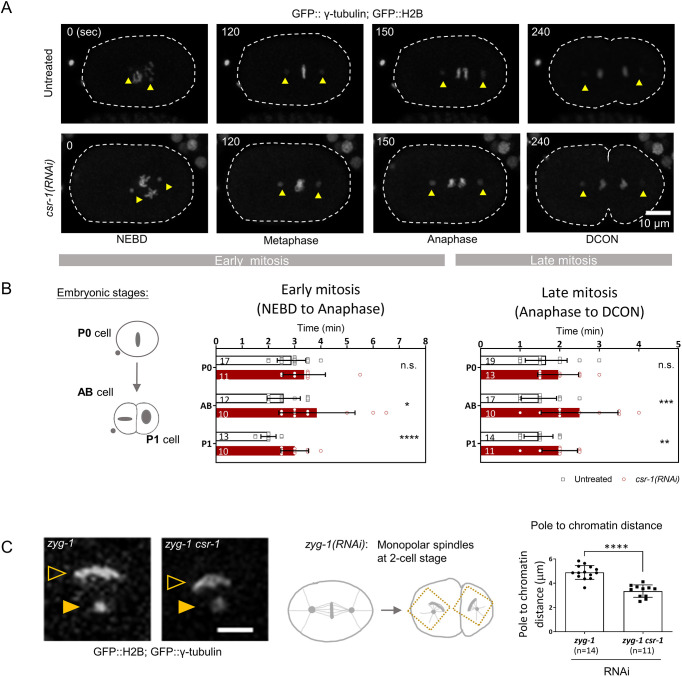
**CSR-1 depletion affects chromosome segregation.** (A) One-cell embryos expressing GFP::γ-tubulin (a marker for spindle poles) and GFP::H2B (a marker for chromatin) were time-lapse imaged to track the spindle poles (yellow arrowheads) and chromatin during cell division. Embryos were either untreated or had RNAi-mediated depletion of CSR-1, as indicated. The times from nuclear envelope breakdown (NEBD) are shown on the top left. Dashed lines mark the embryo outlines. Scale bar: 10 μm. DCON: DNA condensation. Early and late mitosis were classified and quantified in B as the time from NEBD to anaphase onset and the time from anaphase onset to DCON in telophase, respectively. (B) Left: diagram showing the P0 cell in one-cell stage, and AB and P1 cells in two-cell stage. Middle and right: the time spent in early (middle) and late (right) mitotic phases by one-cell embryos (P0 cell) and two-cell embryos (AB and P1 cells) was quantified for untreated and *csr-1*(RNAi) embryos by live-cell imaging as shown in A. The *n* values shown in the graphs represent the number of embryos analyzed. Data are presented as mean±s.d. *P<0.05; **P<0.01; ***P<0.001; ****P<0.0001; n.s., not significant (two-tailed unpaired *t*-test). (C) *zyg-1*(RNAi) inhibits centriole duplication and generates a monopolar spindle at the two-cell stage. Live-cell imaging (left) shows the congressed chromatin mass around the sole spindle pole in *zyg-1*(RNAi) cells expressing GFP::γ-tubulin and GFP::H2B, with or without *csr-1*(RNAi) co-depletion as indicated. The distance between the congressed chromatin (open yellow arrowhead) and the spindle pole (filled yellow arrowhead) was quantified (right). Diagram (middle) shows a schematic of the monopolar spindles at two-cell stage in a *zyg-1*(RNAi) embryo. Data are presented as mean±s.d. *n* values represent the number of monopolar spindle cells analyzed. ****P<0.0001 (two-tailed unpaired *t*-test). Scale bar: 5 μm.

Aneuploidy and missegregation can imply earlier mitotic defects in chromosome–microtubule attachments. Unattached kinetochores will activate the spindle assembly checkpoint and delay anaphase onset, whereas merotelic attachments result in less disturbance to anaphase onset, but the affected cells take longer to resolve the bridged chromatin (Essex et al., 2009; Cimini, 2007). To see whether *csr-1*(RNAi) cells took longer to divide, we measured the time needed for mitotic division in the first two rounds of cell divisions, using GFP-tagged H2B and γ-tubulin to label chromatin and spindle poles, respectively (Fig. 1A). Mitotic phases were divided into early mitosis (time from prophase, when the nuclear envelope breaks down, to anaphase) and late mitosis (time from anaphase to telophase). In the first embryonic division, the *csr-1*(RNAi) embryonic founder cell (P0) showed no significant difference in division time relative to the division time of the untreated control (Fig. 1B). In two-cell embryos with CSR-1 depletion, both early and late mitosis were prolonged when compared to mitosis in the untreated control. Early mitosis was 50% longer than that of the untreated control for both AB and P1 cells. Late mitosis was 70% longer for AB cells and 40% longer for P1 cells following CSR-1 depletion, as compared to that of the untreated control (Fig. 1B). Inter-spindle pole distance was also tracked using GFP::TBG-1 (also referred to here as GFP::γ-tubulin) in one-cell embryos (Fig. 1A, yellow arrowheads). At anaphase onset, chromosome-to-pole movement is minimal but inter-spindle pole distance increases (Espeut et al., 2012; Oegema et al., 2001). In the *csr-1*(RNAi) one-cell embryos, the spindle pole separation was not significantly different from that of the control, despite lagging chromosomes being observed in anaphase (Fig. S1E; Movies 1 and 2).

Prolonged mitosis can occur when chromosomes are misattached to the microtubules (Gregan et al., 2011). To test whether there are additional effects caused by CSR-1 depletion on chromosome dynamics independent of merotelic attachment, we generated a scenario devoid of tension caused by pulling forces from opposite poles. ZYG-1 is a kinase that regulates centrosome duplication (O'Connell et al., 2001). Without ZYG-1, centrosomes fail to duplicate after the first division (Powers et al., 2004). As a result, only one spindle pole remained in AB and P1 cells when we depleted ZYG-1 using RNAi [*zyg-1*(RNAi)]. Spindle pole to chromatin distance was tracked using GFP::γ-tubulin and GFP::H2B in two-cell embryos. Condensed chromosomes at late prometaphase progressively formed an arc shape surrounding the only spindle pole in each of the cells at the two-cell stage, with no tension on the chromosomes (Fig. 1C). In *zyg-1*(RNAi) cells, the average distance between the spindle pole and the chromatin was 4.891 μm*.* Cells with depletion of both ZYG-1 and CSR-1 [*zyg-1 csr-1*(RNAi)] cells had a shorter average chromatin-to-pole distance of 3.349 μm, which was 68.5% of that observed for the *zyg-1*(RNAi) cells (Fig. 1C). Thus, even in a scenario devoid of tension and potential merotelic attachments, chromosome positioning in *csr-1*(RNAi) cells was different from that in cells without CSR-1 knockdown.

### Levels of centromere and kinetochore proteins on chromatin are increased in *csr-1*(RNAi) embryos

**Fig. 2.**
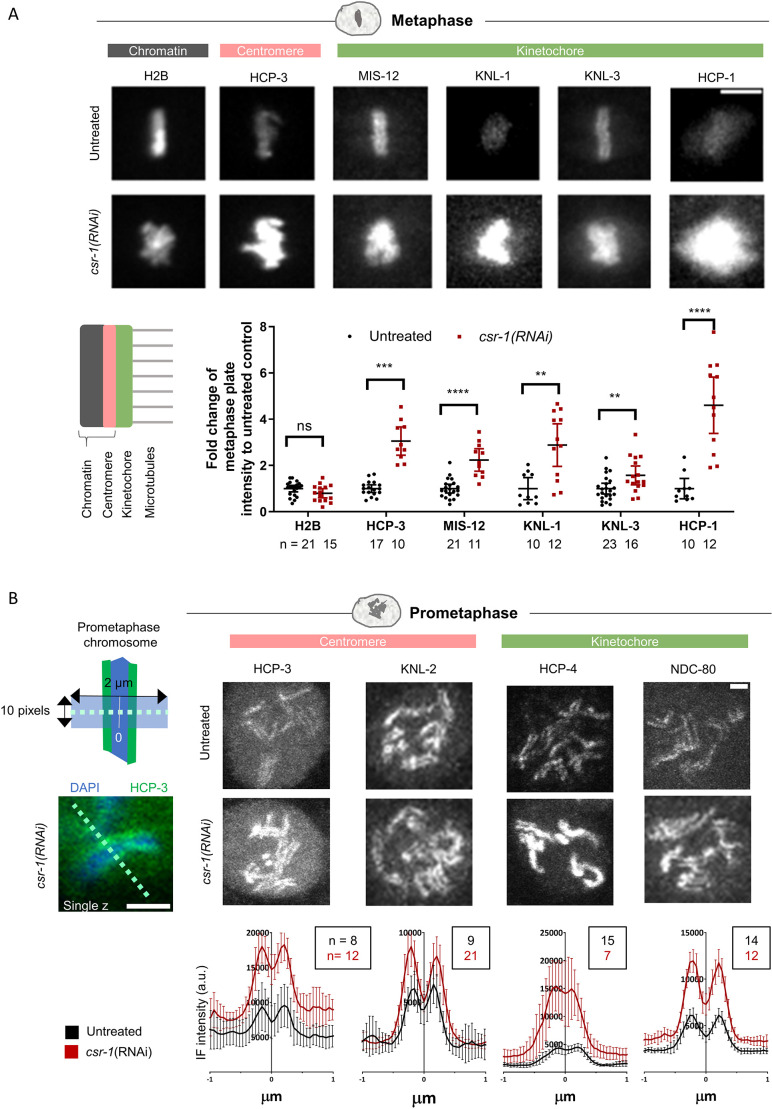
**CSR-1 depletion affects kinetochore and centromere protein localization.** (A) Maximum-projected live-cell images of metaphase chromosomes stained for the indicated proteins in untreated and *csr-1*(RNAi) one-cell embryos (top). Scale bar: 5 μm. Bottom-left: diagram shows a schematic of the constitutive centromere proteins (pink) on chromatin, and the kinetochore components (green), which make attachments to microtubules. Bottom-right: fluorescence intensities on chromatin of different fluorescently tagged kinetochore proteins, as well as histone H2B and HCP-3, were quantified as described in Fig. S2B and in the Materials and Methods. Data are presented as mean±95% confidence interval. The *n* values shown represent the number of embryos analyzed. **P<0.01; ***P<0.001; ****P<0.0001; ns, not significant (two-tailed unpaired *t*-test). (B) Immunofluorescence images and quantification of prometaphase chromosomes from a single plane in untreated and *csr-1*(RNAi) embryos. Fixed embryos at the same cell stage were used to compare the protein distribution and level on condensed chromosomes between untreated control and *csr-1*(RNAi) embryos (HCP-3, two-cell stage; KNL-2, two-cell stage; HCP-4, one-cell stage; NDC-80, two-cell stage). The mid-point between the two peaks was taken as the center (0 μm) to align the line scan intensity profiles of different chromosomes. Representative images of embryos stained for the indicated proteins and the corresponding quantifications of immunofluorescence (IF) intensity are shown on the right. Data are presented as mean±95% confidence interval (a.u., arbitrary units). The *n* values shown represent the number of chromosomes analyzed. Line scan profiles were generated and quantified as described in the Materials and Methods section. The diagram on the top left shows the alignment of line scan measurements. The image on the bottom left shows an example of a chosen chromosome and a 2 µm dashed line drawn along the chromosome width. Scale bars: 1 μm.

Previous studies have provided conflicting evidence regarding the effects of CSR-1 depletion on the chromatin localization of HCP-3. To determine how CSR-1 depletion affects centromere and kinetochore protein occupancy, we quantified protein chromatin localization in live and fixed embryos. Embryos expressing a fluorescently tagged centromere or kinetochore protein were imaged. The metaphase plate appeared bulkier in *csr-1*(RNAi) embryos, likely due to imperfect chromosome alignment (Claycomb et al., 2009; Gerson-Gurwitz et al., 2016). The metaphase plate intensity of histone H2B level was not changed, whereas the metaphase plate intensities of the centromere protein HCP-3 and the kinetochore proteins MIS-12, KNL-1, KNL-3 and HCP-1 were increased significantly in *csr-1*(RNAi) embryos (Fig. 2A).

During prometaphase, centromere and kinetochore proteins became visible on the poleward faces of the chromosomes. We immunostained and selected prometaphase cells for analysis. For all centromeric and kinetochore proteins we tested, the localization remained concentrated on the poleward faces of the chromosomes in *csr-1*(RNAi) embryos, which is consistent with observations of KNL-2 and HCP-3 localization reported previously (Gerson-Gurwitz et al., 2016). Among the proteins quantified (Fig. 2B), the levels of the centromeric protein HCP-3 and the kinetochore proteins HCP-4 and NDC-80, but not of the centromere licensing factor Mis18BP1 homolog KNL-2, increased at the poleward faces of the chromosomes in *csr-1*(RNAi) embryos.

**Fig. 3.**
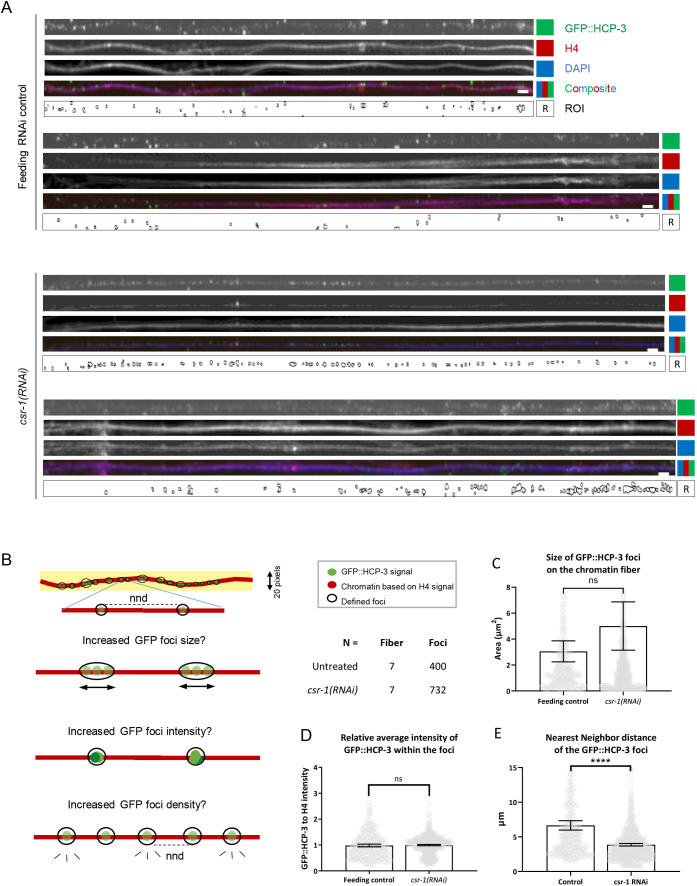
**Distribution of GFP::HCP-3 foci on stretched chromatin fibers.** (A) Representative images of GFP::HCP-3-containing chromatin fibers showing the change of chromatin HCP-3 distribution upon CSR-1 depletion. Stretched chromatin fibers were prepared from the feeding RNAi control and *csr-1*(RNAi) embyro lysates. The chromatin fibers were immunostained to detect GFP (GFP::HCP-3) and histone H4, and DNA was labeled with DAPI. Regions of GFP::HCP-3 foci were defined (ROI) using the GFP::HCP-3 channel. Scale bars: 10 μm. Additional chromatin fiber images are shown in Fig. S3. (B) Diagram explaining how the GFP::HCP-3 foci on the chromatin fibers were characterized in this study. Chromatin fibers were selected using 20-pixel-wide line (yellow background) drawn across the length of fiber (red). A GFP intensity threshold was applied (see Fig. S2C and Materials and Methods) to define the GFP::HCP-3 foci (ROI) based on size and circularity filters. Brighter GFP::HCP-3 on stretched chromatin in this analysis can be resolved into regions of GFP foci that appear either brighter (increased intensity), bigger (increased area) or denser (reduced distance to nearby foci; measured as nearest neighbor distance, nnd). The *n* values for quantification are shown. (C–E) Properties of the GFP::HCP-3 foci were quantified, including the (C) foci size, (D) foci average intensity and (E) nearest neighbor distance (NND), which is inversely correlated to the foci density. The GFP::HCP-3 foci intensities were normalized with the corresponding histone H4 intensities. Data are presented as mean±95% confidence interval. Feeding RNAi control, *n*=400 foci from seven fibers; *csr-1*(RNAi), *n*=723 foci from seven fibers. ****P<0.0001; ns, not significant (two-tailed unpaired *t*-test).

Chromatin was stretched into fibers, based on a previously reported method (Kyriacou and Heun, 2018), to examine the distribution of HCP-3. Consistent with the results of our *in vivo* imaging, *csr-1*(RNAi) chromatin fibers contained more GFP::HCP-3 staining (Fig. 3A). Areas of GFP foci on the chromatin fiber were defined after thresholding and dot clustering (see Fig. S2C and Materials and Methods). Foci areas and the mean intensity of foci on the *csr-1*(RNAi) fibers were not significantly different from those of fibers from the feeding RNAi control (Fig. 3B–E). The density of the foci was estimated by calculating the nearest neighbor distance (NND), which represents the shortest distance for a given HCP-3 focus to its neighboring foci. The NND was lower on the *csr-1*(RNAi) fibers than on fibers from the feeding RNAi control (58% of the control; Fig. 3E). Thus, HCP-3 has increased chromatin occupancy as distinct foci with higher density in *csr-1*(RNAi) embryos.

### HCP-3 mRNA level, protein level and protein dynamics on chromatin remain unchanged in *csr-1*(RNAi) embryos

**Fig. 4.**
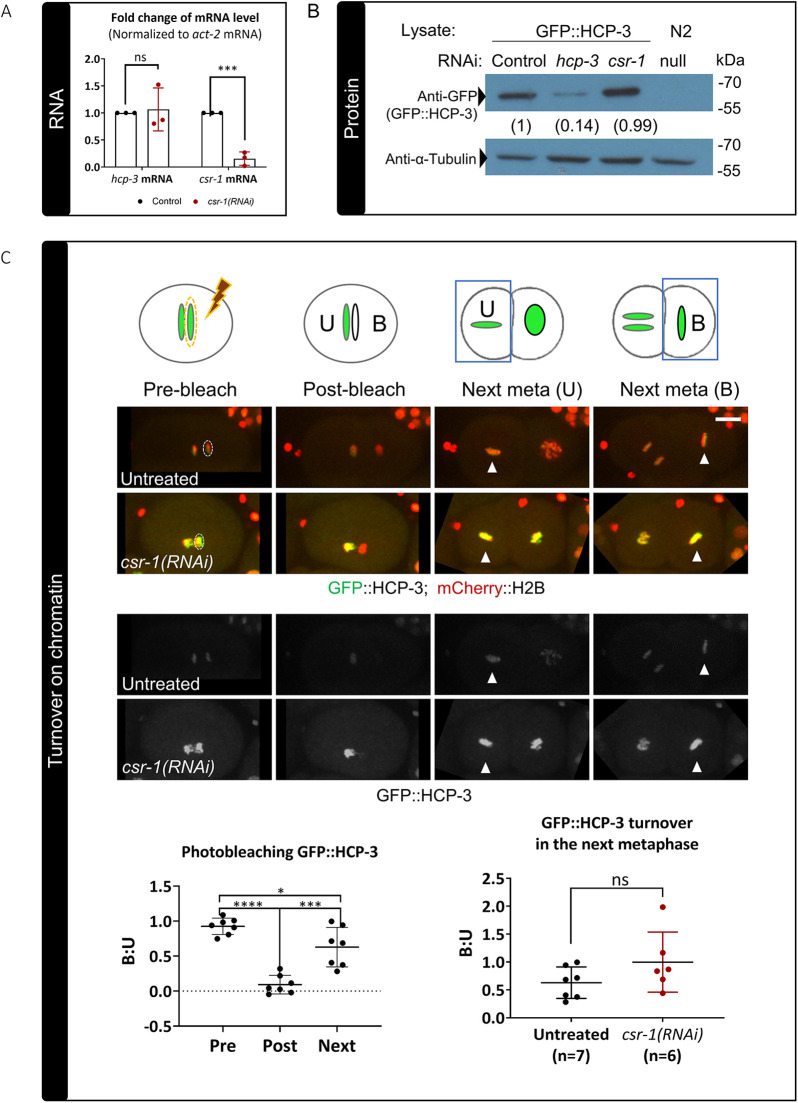
**Effect of CSR-1 depletion on HCP-3 expression and stability on chromatin.** (A) Relative fold change of *hcp-3* and *csr-1* mRNA levels in the *csr-1*(RNAi) embryos as compared to the feeding RNAi control. The mRNA levels are normalized to those of *act-2* mRNA*.* Three biological replicates were performed, and the data are presented as mean±s.d. ***P<0.001; ns, not significant (two-tailed unpaired *t*-test). (B) Immunoblotting shows that the GFP::HCP-3 protein level remains unchanged in *csr-1*(RNAi) embryos as compared to levels in the feeding RNAi control. Anti-α-tubulin is shown as a loading control. N2: wild-type lysate, biological control without GFP::HCP-3. Numbers below the anti-GFP blot indicate the mean normalized GFP::HCP-3 level relative to the feeding RNAi control from three biological replicates (Fig. S4A). Positions of molecular mass markers are indicated on the right. (C) Top: diagram and representative fluorescence images showing the photobleaching assay used to investigate HCP-3 chromatin turnover in untreated and *csr-1*(RNAi) embryos expressing GFP::HCP-3 (green) and mCherry::H2B (red). GFP::HCP-3 on one of the anaphase sister chromatids was photobleached using a high-power laser with hand-drawn ROI (dashed ellipses), resulting in an unbleached (U) chromatid and a bleached (B) chromatid. In the next metaphase (next meta), the fluorescence intensities of the two daughter cells were measured (white arrowheads). The intensity ratio of the GFP signal for bleached over unbleached chromatin (B:U) was measured to quantify protein turnover on chromatin. The reappearing GFP signal on the bleached chromatin represents newly recruited GFP::HCP-3. Scale bar: 10 μm. Bottom left: quantification of the B:U ratio of the chromatin GFP::HCP-3 signal in untreated control embryos before (pre) and after (post) photobleaching in the first anaphase and in the next metaphase (next). Bottom right: the B:U ratio for untreated control and *csr-1*(RNAi) embryos in the next metaphase. Data are presented as mean±s.d. The *n* values shown represent the number of photobleached embryos analyzed. *P<0.05; ***P<0.001; ****P<0.0001; ns, not significant (two-tailed unpaired *t*-test).

We observed a change in chromatin localization of centromeric and kinetochore proteins following knockdown of CSR-1; however, the expression levels of the corresponding genes are not altered in the *csr-1* hypomorph transcriptome (Fig. S4C) (Claycomb et al., 2009; Gerson-Gurwitz et al., 2016). We verified HCP-3 mRNA and protein levels in the embryo lysate after CSR-1 knockdown. Feeding RNAi was performed to collect a sufficient number of knockdown embryos for reverse transcription–quantitative PCR (RT-qPCR) and western blotting. The GFP::HCP-3 strain used contains a sole copy of GFP-fused HCP-3 generated by *Mos1*-mediated single-copy insertion (*mos*SCI) in a *hcp-3(ok1892)* deletion background (Gassmann et al., 2012). In *csr-1*(RNAi) embryos, *hcp-3* mRNA level was not significantly different from that in the feeding RNAi control (Fig. 4A) and the total embryonic GFP::HCP-3 protein level was not significantly different from that in the feeding RNAi control (Fig. 4B; Fig. S4A). This indicated that the increased chromatin localization we observed is not a result of increased HCP-3 expression.

Another possibility is that the chromatin-assembled HCP-3 stays on chromatin longer, resulting in increased HCP-3 on chromatin at a given time. CENP-A is inherited in a semi-conservative manner in humans and *Drosophila*, as new CENP-A is loaded during each cell cycle to replenish replicated DNA (Jansen et al., 2007; Mellone et al., 2011). In contrast, *C. elegans* HCP-3 does not stay on chromatin and turns over almost completely in one cell cycle (Gassmann et al., 2012). To assay HCP-3 chromatin stability, GFP::HCP-3 on one of the anaphase sister chromatids in a one-cell stage embryo was photobleached using a 488 nm laser. We measured the GFP intensity on the next metaphase plates in the daughter cells containing the photobleached and the unbleached chromatin, respectively. The recovered GFP signal on the photobleached chromatin represents new GFP::HCP-3 loaded within one cell cycle. In untreated embryos, the GFP intensity was observed to be recovering during telophase as the nuclear envelope reformed. The ratio of GFP on the bleached chromatin to GFP on the unbleached chromatin in the next metaphase did not drop in *csr-1*(RNAi) embryos when compared to that in the untreated control (Fig. 4C). Therefore, there was no detectible decrease in GFP::HCP-3 turnover on chromatin, and thus a change in dynamics cannot explain the observed increase in HCP-3 on chromatin.

### The increase in HCP-3 on chromatin in *csr-1*(RNAi) embryos is not completely dependent on licensing factor KNL-2 and loading factor LIN-53

**Fig. 5.**
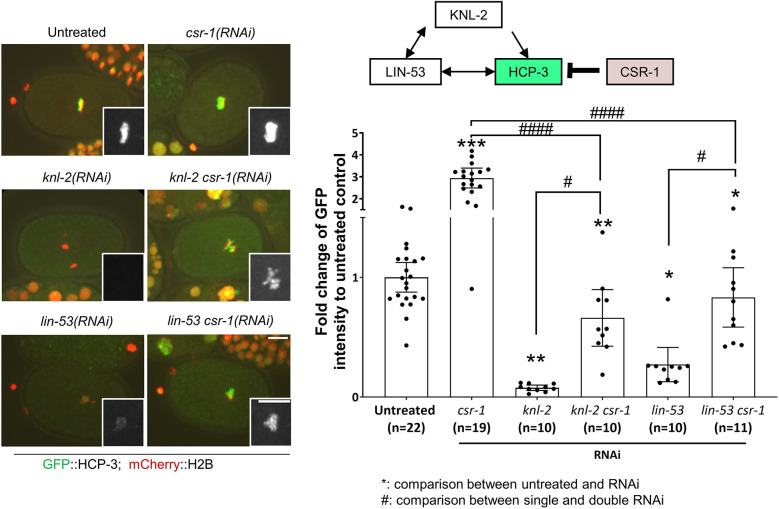
**Depletion of CSR-1 promotes HCP-3 chromatin localization in embryos depleted of HCP-3 loading factors.** Representative images and quantification of the mean GFP::HCP-3 intensity of the metaphase plate in one-cell embryos with RNAi treatments for depletion of CSR-1, KNL-2 and LIN-53 alone or in combination, as indicated. Inset images show magnified views of the GFP::HCP-3 signal on the metaphase plate. Diagram shows the localization dependency relationship among the proteins. Scale bars: 10 μm; insets, 5 μm. Data are presented as mean±95% confidence interval. The *n* values shown represent the number of one-cell embryos analyzed. *P<0.05; ^#^*P*<0.05; **P<0.01; ***P<0.001; ^####^*P*<0.0001 (two-tailed unpaired *t*-test). The representative images of untreated control, *knl-2 csr-1*(RNAi) and *lin-53 csr-1*(RNAi) are also shown in Fig. S6A for comparison with PRG-1-depleted embryos as part of the same experiment.

As we observed no effect of CSR-1 knockdown on HCP-3 expression and turnover on chromatin, we therefore propose that the increase in HCP-3 on chromatin in *csr-1*(RNAi) embryos is caused by an enhanced recruitment of HCP-3 onto the centromeric chromatin. HCP-3 localization requires KNL-2 and LIN-53 (Maddox et al., 2007; Lee et al., 2016). To determine whether the additional deposition of HCP-3 after CSR-1 knockdown occurs through KNL-2 or LIN-53, we depleted CSR-1 in embryos with RNAi-mediated depletion of KNL-2 [*knl-2*(RNAi)] or LIN-53 [*lin-53*(RNAi)]. Noticeable GFP::HCP-3 was seen on the dense chromatin mass in embryos with depletion of both KNL-2 and CSR-1 [*knl-2 csr-1*(RNAi)], with an intensity that was 6.5-fold that of the corresponding GFP::HCP-3 signal in *knl-*2(RNAi) embryos, whereas the GFP::HCP-3 intensity observed in embryos with depletion of both LIN-53 and CSR-1 [*lin-53 csr-1* (RNAi)] was 3.3-fold that observed in *lin-*53(RNAi) embryos (Fig. 5; Fig. S6C). However, the GFP::HCP-3 intensity observed for both of the double RNAi lines was lower than that in *csr-1*(RNAi) embryos. These results suggest that in *csr-1*(RNAi) embryos, some HCP-3 can be loaded onto the chromatin by mechanisms that are independent of KNL-2 or LIN-53. Thus, the increased deposition of HCP-3 in *csr-1*(RNAi) embryos is not completely hindered by the depletion of KNL-2 or LIN-53. Double knockdown of another Argonaute with KNL-2 or LIN-53 did not result in an increase in the chromatin-bound levels of HCP-3 (Fig. S6), suggesting that the effect is specific to CSR-1.

Taken together, our data show that HCP-3 localization on chromatin is increased in *csr-1*(RNAi) embryos. There was no change in the total embryonic expression of HCP-3, and there was no apparent reduction in HCP-3 protein turnover on the chromatin. The increase in HCP-3 localization was observed on chromatin fibers where foci were far enough apart to be resolved. Such ectopic localization might be driven by a non-classical pathway that is normally suppressed by CSR-1.

### The CSR-1 RNAi pathway and CSR-1 slicer activity are important for regulating levels of HCP-3 on chromatin

**Fig. 6.**
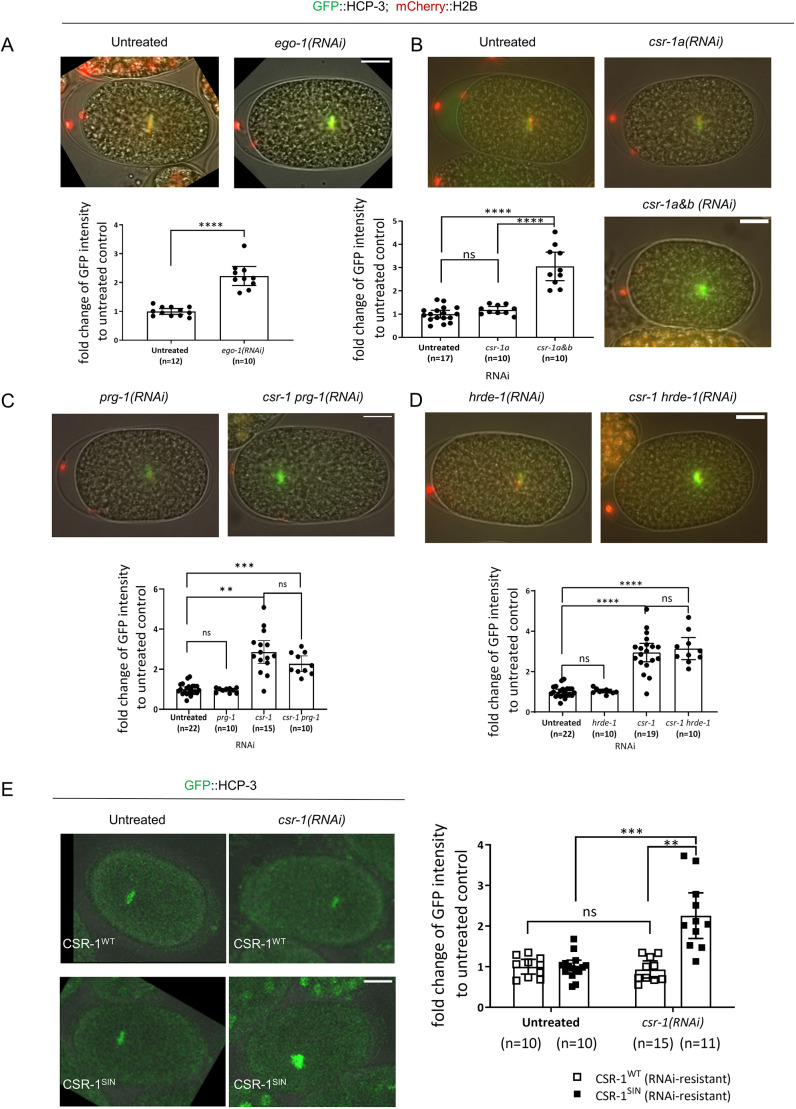
**Requirements for repression of HCP-3 chromatin localization by CSR-1.** Representative images and quantification of the mean GFP::HCP-3 intensity of the metaphase plate in one-cell embryos with (A) knockdown of the RNA-dependent RNA polymerase of the CSR-1 RNAi pathway, EGO-1 [*ego-1*(RNAi)]; (B) isoform-specific knockdown of CSR-1A [*csr-1a*(RNAi)], compared to pan-CSR-1 depletion [*csr-1a&b*(RNAi)]; (C) knockdown of PRG-1, an Argonaute of the germline piRNA pathway, either alone [*prg-1*(RNAi)] or in combination with CSR-1 depletion; (D) knockdown of HRDE-1, an Argonaute of another germline RNAi pathway, either alone [*hrde-1*(RNAi)] or in combination with CSR-1 depletion; and (E) knockdown of endogenous CSR-1 in strains expressing transgenic, RNAi-resistant versions of either wild-type CSR-1 (CSR-1^WT^) or a slicer-inactive mutant (CSR-1^SIN^). Scale bars: 10 μm. GFP intensity is shown relative to the levels in the untreated controls. Data are presented as mean±95% confidence interval. The *n* values shown represent the number of one-cell embryos analyzed. **P<0.01; ***P<0.001; ****P<0.0001; ns, not significant (two-tailed unpaired *t*-test).

To identify factors important for regulation of chromatin HCP-3 by CSR-1, we tested different RNAi pathways, CSR-1 isoforms and slicer activity. CSR-1-bound siRNA are synthesized by the RNA-dependent RNA polymerase EGO-1 (Claycomb et al., 2009; Seth et al., 2013). Knocking down EGO-1 resulted in 2-fold increase in GFP::HCP-3 intensity at the metaphase plate compared to that in the untreated control (Fig. 6A). Thus, EGO-1, and possibly the siRNA it synthesizes, are also required for HCP-3 loading on chromatin.

Two CSR-1 isoforms have different expression patterns and bind different set of siRNAs (Nguyen and Phillips, 2021). The larger isoform CSR-1A is more abundant in soma and sperm cells than CSR-1B, which is enriched in germline cells (Claycomb et al., 2009; Charlesworth et al., 2021). To determine whether a specific isoform of CSR-1 regulates HCP-3 localization, we knocked down the longer isoform CSR-1A specifically by targeting the first exon of *csr-1a*, which is unique for this isoform (Fig. S5D). We validated the *csr-1a* knockdown by using RT-qPCR, finding that the mRNA level was 12.23% of that in untreated embryos (Fig. S5B). Depletion of CSR-1A resulted in a chromatin GFP::HCP-3 level that was 1.3-fold of that in the untreated control (Fig. 6B), much lower than the level observed upon depletion of both isoforms, which was 3-fold that of the untreated control. It is not possible to knock down CSR-1B alone due to the overlapping sequences of the two isoforms. From our results, the increase in GFP::HCP-3 was not caused by depletion of CSR-1A, but was probably mostly caused by depletion of CSR-1B.

Next, we checked whether CSR-1 regulates HCP-3 by antagonizing other germline pathways. CSR-1 is antagonistic to PRG-1 Argonaute, which interacts with piRNAs in the germline, and HRDE-1 Argonaute, which binds the secondary siRNA generated to silence genes (Wedeles et al., 2013b; Youngman and Claycomb, 2014). To determine whether the observed increase in GFP::HCP-3 after CSR-1 depletion is affected by PRG-1 or HRDE-1, we depleted CSR-1 in embryos deficient for either PRG-1 [*csr-1 prg-1*(RNAi)] or HRDE-1 [*csr-1 hrde-1*(RNAi)]. We expected that if these pathways are responsible for the HCP-3 increase, then levels of HCP-3 on chromatin in *csr-1 prg-1*(RNAi) and *csr-1 hrde-1*(RNAi) embryos would return to those seen in the untreated control. However, co-depleting CSR-1 with either PRG-1 or HRDE-1 did not prevent the increase in HCP-3 chromatin localization. The GFP::HCP-3 intensities in both *csr-1 prg-1*(RNAi) and *csr-1 hrde-1*(RNAi) remained ∼3.4-fold that the untreated control (Fig. 6C,D). This indicates that the HCP-3 localization increase observed in *csr-1*(RNAi) embryos does not depend on the PRG-1 and HRDE-1 RNAi pathways.

It has been previously demonstrated that CSR-1 cleaves mRNAs (Aoki et al., 2007). The slicing activity lies within the ‘DDH’ motif of the PIWI domain, which binds divalent magnesium ions (Liu et al., 2004; Schwarz et al., 2004). A CSR-1 slicing-inactive mutant (CSR-1^SIN^), carrying point mutations D606A and D681A, induces chromosome missegregation (Yigit et al., 2006; Aoki et al., 2007; Gerson-Gurwitz et al., 2016). To determine whether the slicing activity of CSR-1 is critical for restricting HCP-3 chromatin localization, we measured GFP::HCP-3 metaphase plate intensity in strains expressing transgenic re-encoded versions of wild-type CSR-1 (CSR-1^WT^) or CSR-1^SIN^ that were resistant to our RNAi treatment against endogenous CSR-1 (Fig. 6E). With the endogenous CSR-1 knocked down, embryos expressing CSR-1^SIN^ showed a significant increase in the GFP::HCP-3 metaphase plate intensity, but this increase was not seen with CSR-1^WT^. Thus, CSR-1-restricted HCP-3 localization requires the PIWI domain slicing activity of CSR-1. In short, we identified that the CSR-1 regulation of HCP-3 localization on chromatin requires EGO-1, isoform CSR-1B and CSR-1 slicer activity.

## DISCUSSION

**Fig. 7.**
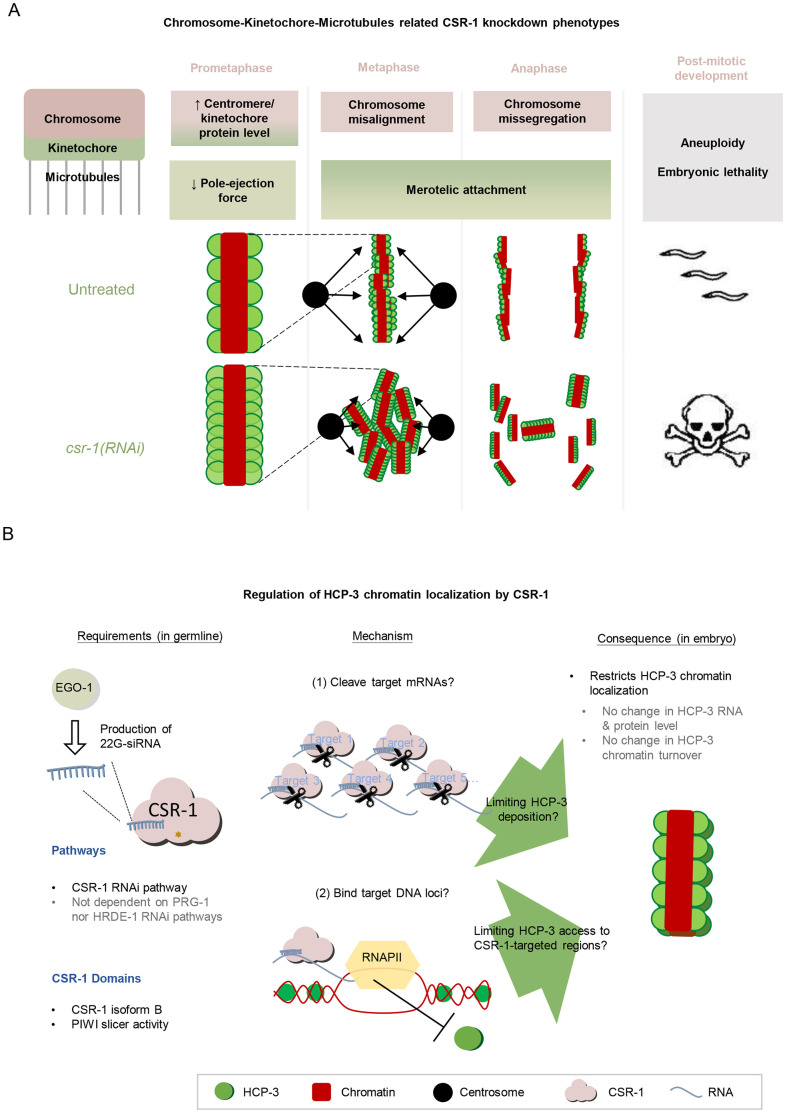
**Chromosome segregation defects in mitosis upon CSR-1 knockdown and summary of how CSR-1 can repress HCP-3 localization on centromeres.** (A) A summary of CSR-1 knockdown phenotypes related to chromosome segregation in mitosis. (B) A proposed mechanism for how CSR-1 affects HCP-3 centromere localization. The germline-enriched CSR-1 isoform B with its PIWI domain is important for regulating embryonic HCP-3 loading onto chromatin. This could be achieved by regulating (1) the level of a target or a subset of mRNA targets by cleaving mRNA, as proposed previously (Gerson-Gurwitz et al., 2016) or (2) an epigenetic mark inhibitory for HCP-3 loading on the target genome via physical interaction with the chromatin in an RNA polymerase II (RNAPII)-dependent manner, as has previously been proposed (Wedeles et al., 2013b). Pathways and CSR-1 domains found to be important for HCP-3 repression are summarized.

We have investigated the role of CSR-1 in chromosome segregation in multiple embryonic cell cycle events (Fig. 7A). In *csr-1*(RNAi) embryos, we observed abnormality in the overall time taken for mitosis. The longer early mitosis suggests defects in the chromosome alignment or activated spindle checkpoint that could delay anaphase onset. The prolonged late mitosis suggests that the microtubule connections to the kinetochore interface are defective in *csr-1*(RNAi) embryos and that the merotelic attachment persists upon chromosome segregation. On the other hand, we observed a shorter chromosome–pole distance in *zyg-1 csr-1*(RNAi) embryos when compared to that in control *zyg-1*(RNAi) embryos. The reduced distance between the chromatin and the spindle pole in *zyg-1 csr-1*(RNAi) embryos reflects a smaller net force to repel chromosome from the spindle pole (Maiato et al., 2017). The change in force could result from a stronger kinetochore-dependent pole attraction, consistent with our observation of increased levels of the kinetochore protein NDC-80 on chromosomes. Alternatively, the change can result from a weaker pole repulsion, consistent with previously reported unstable spindles (Gerson-Gurwitz et al., 2016). Kinetochore–microtubule misattachment, especially merotelic attachment where one kinetochore is attached to microtubules from both spindle poles, can lead to chromosome missegregation. Merotelic attachments are resolved by Aurora B kinase AIR-2 to grant the cell another chance for re-connection (Kaitna et al., 2002). However, we did not observe much change in AIR-2 localization in CSR-1 knockdown embryos (Fig. S1F), but it remains possible that AIR-2 activity is altered.

We examined the chromosome–microtubule interface by quantifying the protein signals on chromosomes. Overall, the level of HCP-3 and kinetochore protein signal on prometaphase chromosomes significantly increased in *csr-1*(RNAi) embryos, while the centromere and kinetochore proteins remain concentrated on the poleward faces on the chromosomes. This is similar to the line scan results reported by Gerson-Gurwitz et al. in their study (Gerson-Gurwitz et al., 2016), where each line scan of HCP-3 signal was normalized to its own peak intensity to study the pattern and distribution of holocentromeres across the transverse plane of the condensed chromosome. By contrast, our analysis focused on the absolute intensity change in *csr-1*(RNAi) embryos rather than the normalized distribution pattern. These increased kinetochore proteins can act as additional anchor points for microtubules binding, thereby changing how chromosomes dynamically move, align and segregate.

With embryo lysis and mechanical stretching, the chromatin signal of the centromere protein HCP-3 was resolved into foci (Fig. 3; Fig. S3), which became denser on fibers from *csr-1*(RNAi) embryos compared to the control fibers. Consistent with our intensity quantification, Ladouceur et al. (2017) have reported brighter HCP-3 signal on the chromosomes in *csr-1*(RNAi) animals, and they also attempted to examine the HCP-3 distribution within the chromatin based on a different method. By squashing metaphase cells, they obtained visually discrete HCP-3 dots that appeared brighter and had no significant change in number in *csr-1*(RNAi) cells. We hypothesized that in our chromatin fiber assay the chromatin was further stretched than by cell squashing, thus we found discrete foci at a higher density in *csr-1*(RNAi) fibers.

In the future, it will be important to determine the specific genomic sequences where HCP-3 binds when CSR-1 is depleted. This would help distinguish between two potential scenarios: (1) the increase in HCP-3 foci occurs specifically within the centromeric regions or (2) there is a broader redistribution and spread of HCP-3 binding to additional genomic regions beyond the centromeres, as observed in other systems when heterochromatin flanking the centromere is disrupted (Scott et al., 2006). However, precisely pinpointing the locations of the holocentromeres on the chromatin fibers is technically challenging in *C. elegans* as the holocentromeres do not form on typical repeat sequences. Future investigation will be needed to fully characterize the changes in HCP-3 localization under these conditions.

Whereas previous work (Ladouceur et al., 2017) and this study have found an increase in HCP-3 localization on chromosomes after CSR-1 depletion based on different methods, Cecere et al. (2014) have reported how the relative HCP-3 localization around CSR-1 targets changes in the absence of CSR-1. Relative to the occupancy of CSR-1 targets (*mes-4* and *pgl-1*), the HCP-3 occupancy in the genes flanking these CSR-1 targets is reduced in a *csr-1* hypomorph strain, as assayed by ChIP-qPCR (Cecere et al., 2014). Alternatively, the ChIP-qPCR results could be interpreted as indicating increased HCP-3 binding to CSR-1 targets, which typically have low HCP-3 occupancy (Gassmann et al., 2012). This alternative interpretation of the lower relative levels of HCP-3 on the regions neighboring CSR-1 targets is consistent with our finding that HCP-3 occupancy on chromatin increases after CSR-1 depletion. In short, our data and the work of others shows that HCP-3 localization increases significantly and the overall distribution on chromatin changes after CSR-1 depletion.

Building on these insights, we further investigated the mechanistic relationship between CSR-1 and HCP-3 localization. HCP-3 is one of the targets of CSR-1 (Claycomb et al., 2009), suggesting a potential regulatory role for CSR-1 in controlling HCP-3 expression. In other organisms, excess CENP-A loading can promote the formation of ectopic centromeres when CENP-A is overexpressed (Tomonaga et al., 2003; Heun et al., 2006; Amato et al., 2009). However, for HCP-3 in *C. elegans*, the observed change was mainly in the amount of protein deposited onto chromatin, rather than changes in HCP-3 mRNA or protein levels. Specifically, neither the HCP-3 mRNA and protein levels, nor its chromatin turnover rate were affected by CSR-1 depletion (Fig. 4). In addition, using a re-encoded version of GFP::HCP-3 strain (Gerson-Gurwitz et al., 2016), we found that the level of GFP::HCP-3 on chromatin still increased 2.8-fold upon CSR-1 depletion compared to levels in the untreated control (Fig. S4B). Therefore, regulation of HCP-3 localization by CSR-1 is not specific to the endogenous *hcp-3* sequence. It is possible that CSR-1 is still able to recognize the re-encoded *hcp-3* sequence. Our results instead imply that normally not all embryonic HCP-3 protein incorporates onto the chromatin, and CSR-1 plays a role in controlling how much embryonic HCP-3 is loaded. We also tried to reduce HCP-3 level using different partial depletion conditions, and this was not sufficient to rescue the chromosome missegregation caused by depletion of CSR-1 (Fig. S5E). Taken together, these findings suggest that the increased HCP-3 localization on chromatin might have caused the observed chromosome segregation defects.

As CSR-1 did not affect HCP-3 localization by directly regulating its transcripts, we further tested multiple perturbations to identify factors that contribute to the increase in GFP::HCP-3 chromatin localization in *csr-1*(RNAi) embryos (Fig. 6). We found that maintaining a normal HCP-3 chromatin level requires CSR-1 slicer activity, as well as expression of the CSR-1B isoform. Specific depletion of the CSR-1A isoform, which is enriched in somatic cells, did not lead to a dramatic elevation of GFP::HCP-3 on chromatin, whereas depletion of both isoforms did. CSR-1A depletion is dispensable for proper chromosome segregation and embryonic viability (Fig. S5C) (Claycomb et al., 2009; Gerson-Gurwitz et al., 2016). Therefore, we believe CSR-1 regulates HCP-3 mainly via the CSR-1B isoform, which is enriched in the hermaphrodite germline. The PIWI domain of CSR-1, which controls target cleavage and binding functions, also appears to play a role in restricting HCP-3 localization. Mutation of the PIWI domain abolishes the ability of CSR-1 to cleave the target (Aoki et al., 2007) and globally affects the abundance and binding profile of CSR-1-associated 22G siRNAs (Singh et al., 2021). Besides impacting transcript regulation, the PIWI domain also interacts with protein cofactors, such as glycine–tryptophan (GW)-repeat proteins, which can repress translation (Liu et al., 2005; Eulalio et al., 2008). Therefore, it is possible that CSR-1 affects HCP-3 localization through PIWI domain-mediated interactions with regulatory factors.

So what is the mechanism by which CSR-1 restricts the chromatin localization of HCP-3? One intriguing possibility is that CSR-1 restricts HCP-3 occupancy indirectly through regulation of the chromatin loading machinery acting on HCP-3 (Fig. 7B). A previous study has reported that CSR-1-targeted genome loci and methylation of histone H3 at lysine 36 are both inversely correlated with HCP-3 occupancy (Gassmann et al., 2012), and there are lines of evidence suggesting that the CSR-1 RNAi pathway can deposit epigenetic marks on the genomic positions of its targets that can propagate to the embryos (Claycomb et al., 2009; Wedeles et al., 2013a,b; Tu et al., 2015). It is possible that the CSR-1 acts *in trans* at multiple loci to exclude HCP-3 binding. For example, CSR-1 has been shown to exhibit transgenerational protection of its targets from piRNA-mediated silencing, likely via creating protective epigenetic marks on the gene loci of its targets (Wedeles et al., 2013b). The germline PRG-1 PIWI RNAi pathway targets exogenous sequences and produce secondary siRNAs that bind the nuclear HRDE-1 Argonaute to mediate gene silencing (Batista et al., 2008; Lee et al., 2012; Seth et al., 2013; Xu et al., 2018; Montgomery et al., 2021). However, our data indicate that neither PRG-1 nor HRDE-1, which are essential for establishing and maintaining gene silencing in the germline, are required for the ability of CSR-1 to suppress HCP-3 localization to chromatin. Future study will be needed to explore whether there are alternative ways that CSR-1 establishes a protective chromatin state to restrict HCP-3 localization.

With its slicer function, CSR-1 might regulate target transcripts important for depositing the centromeric histone variant HCP-3 onto chromatin. Recruitment of HCP-3 onto the holocentromere is interdependent on KNL-2 and partially dependent on LIN-53 in embryos (Maddox et al., 2007; Lee et al., 2016). However, the expression levels of KNL-2 and LIN-53 are unchanged in CSR-1-depleted embryos and in a CSR-1 PIWI-domain mutant (Claycomb et al., 2009; Gerson-Gurwitz et al., 2016). The persistent HCP-3 localization observed in *knl-2 csr-1*(RNAi) embryos raises the possibility of HCP-3 loading through alternative mechanisms when CSR-1 is depleted. Potential candidates include histone chaperones or other histones. For example, CSR-1 might suppress HCP-3 localization by limiting the expression of histone chaperones that load HCP-3 onto the chromosomes. Additionally, CSR-1 has been shown to slice the pre-mRNA of canonical histone mRNAs to promote their maturation (Avgousti et al., 2012). By altering the level of mature histone H3 and histone H4 mRNAs, CSR-1 might indirectly affect the incorporation of HCP-3, as the histone H3:H4 ratio has been shown to impact localization of the CENP-A homolog Cnp-1 in fission yeast (Castillo et al., 2007).

Interestingly, the increase in HCP-3 chromatin localization in *csr-1*(RNAi) embryos does not appear to be directly associated with the chromosome alignment defects at metaphase. Reducing the levels of the kinesin KLP-7 in *csr-1*(RNAi) embryos rescues the microtubule assembly defect and improves chromosome alignment at the metaphase plates. However, KLP-7 depletion does not rescue the chromosome missegregation phenotype in *csr-1*(RNAi) embryos (Gerson-Gurwitz et al., 2016). Furthermore, we observed that the intensity of GFP::HCP-3 in *csr-1 klp-7*(RNAi) double knockdown embryos was similar to that in *csr-1*(RNAi) embryos (Fig. S5F). This suggests that the increase in HCP-3 chromatin localization caused by *csr-1*(RNAi) is not directly linked to the defects in microtubule assembly or chromosome alignment. Additionally, eliminating potential merotelic attachment in *zyg-1*(RNAi) embryos was not sufficient to make the forces on chromatin position equal between CSR-1 depletion and the control condition. Therefore, CSR-1 appears to ensure proper chromosome movement in the embryos through multiple mechanisms, including maintaining microtubule stability, preventing over-assembly of centromere and kinetochore proteins, and preventing merotelic kinetochore attachment.

Here, we have shown evidence that chromosome missegregation in CSR-1-depleted embryos can be caused by a misregulated HCP-3 chromatin level, which in turns changes the kinetochore–microtubule interface and potentially increases the frequency of merotelic attachment, leading to chromosome missegregation. Our study has shed light on how the deposition of centromeric proteins is regulated in *C. elegans* and has exemplified how an RNAi pathway can be involved in the determination of centromere regions in organisms containing non-regional centromeres. It will be interesting to investigate whether Argonautes and RNAi pathways have conserved functions in maintaining centromeres or controlling CENP-A loading across species. In humans, the AGO2 RNAi pathway is also required for proper chromosome segregation, with depletion of AGO2 or a slicer-inactive AGO2 mutant leading to abnormal α-satellite RNA levels and CENPC1 mislocalization to the chromosome arms (Huang et al., 2015). Furthermore, it will be important to explore the potential contribution of epigenetic modifications and CSR-1-genome interactions in restricting HCP-3 localization. Even though humans and *C. elegans* have different centromere architectures and DNA sequences, the epigenetic regulation, including histone modifications, RNAi pathways, transcription and non-coding RNAs, might be conserved.

## MATERIALS AND METHODS

### Worm maintenance and harvesting

Hermaphrodites were maintained at room temperature (20°C) and fed on OP50 *E. coli* on MYOB plates (3.1 g/l Bacto Peptone, 2 g/l NaCl, 0.55 g/l Tris-HCl, 0.24 g/l Tris base, 8 mg/l cholesterol, 20 g/l agar). Worms were collected from agar plates or from liquid culture in M9 buffer. Embryos were harvested from hypochlorite-treated gravid adults [1% hypochlorite, 0.5 M NaOH or KOH in M9 buffer (see below)]. Lysates were incubated on ice for 10 min until most embryos were released. Embryos were collected by centrifuging at 700–800 ***g*** for 1 min followed by rinsing in M9 buffer (22 mM KH_2_PO_4_, 42.3 mM Na_2_HPO_4_, 2 mM MgSO_4_ and 85.6 mM NaCl). Strains used in this study are listed in Table S1. Worm strains with OD prefix were generous gifts from the lab of Dr Arshad Desai and Dr Karen Oegema, University of California, San Diego (UCSD), USA.

### RNAi

RNAi knockdown was carried out mainly by injecting dsRNA, if not stated otherwise. Other knockdown methods included feeding and soaking RNAi.

#### RNAi by injection

L4 larvae or young adults were injected as described previously (Lin and Yuen, 2021) with 1–1.5 μg/μl dsRNA of the target genes. For double knockdown, dsRNAs were mixed in a 1:1 ratio. For partial *klp-7* RNAi, one part of *klp-7* dsRNA was mixed with 16 parts of Milli-Q water or with *csr-1* dsRNA as described previously (Gerson-Gurwitz et al., 2016)*.* Injected worms were incubated on MYOB agar plates with OP50 *E. coli* for 24 h at 20–25°C before imaging or experimental procedures.

#### RNAi by feeding

Feeding RNAi was used when large number of embryos was needed (Fig. 3 and Fig. 4A,B). L4 larvae were fed with HT115 *E. coli* bacteria carrying a vector expressing either null, *hcp-3* or *csr-1* dsRNA for 24–36 h at 20°C. The negative RNAi control was HT115 bacteria carrying empty PL4440 vector (a gift from the lab of Dr Garry Wong, University of Macau, Macau, China). The bacterial strain expressing *csr-1* dsRNA was ordered from Dharmacon (CeRNAi Feeder F20D12.1). The *hcp-3* dsRNA vector was generated from PL4440 (WYYp142). A 50 ml overnight bacterial culture was diluted 1:50 and then incubated for 3 h at 37°C followed by 4 h IPTG (4 mM) induction. The induced bacteria were concentrated 10-fold then spiked with IPTG (4 mM), ampicillin (50 μg/ml) and tetracycline (10 μg/ml) before seeding onto a MYOB agar plate. Next, 5 μl of L4 larva pellet was added onto each plate with dsRNA-expressing bacteria or PL4440-containing bacteria (feeding RNAi control). For each treatment, ∼10–15 plates were used for western blotting or cDNA preparation.

The RNAi knockdown efficiency was verified by embryonic lethality test (Fig. S5A). For those genes with no embryonic lethality upon knockdown, the knockdown efficiency was verified by RT-qPCR instead (Fig. S5B).

#### Double-stranded RNA production

For dsRNA production, 800–1000 base pair coding regions of targeted genes were amplified by PCR from N2 *C. elegans* cDNA or genomic DNA using primers listed in Table S2. Purified PCR products were subjected to *in vitro* transcription (MEGAscript T3/T7 Transcription Kit; Life Technologies). Reaction products were digested with TURBO DNase at 37°C for 15 min and purified (MEGAclear Kit; Life Technologies). Eluates were incubated at 68°C for 10 min followed by 37°C for 30 min to generate dsRNA.

### Immunofluorescence

After dissection of gravid hermaphrodites, embryos were freeze-cracked in liquid nitrogen, fixed in methanol at −20°C for 30 min, rehydrated in phosphate-buffered saline (PBS) for 5 min and blocked in AbDil (4% BSA and 0.1% Triton X-100 in PBS) at room temperature for 20 min. Incubation of primary antibody was performed at 4°C overnight (list of antibodies and conditions in Table S3). Slides were washed with 0.5% Tween 20 in PBS (PBST) before fluorescence-conjugated secondary antibody (1:500; Jackson ImmunoResearch Laboratories) incubation at room temperature for 1 h. Samples was incubated with DAPI for 5 min and mounted (ProLong Diamond Antifade mountant; Life Technologies). Dr Arshad Desai (UCSD, USA) gifted the anti-AIR-2, anti-HCP-4, anti-KNL-2 and anti-NDC-80 antibodies.

### Image acquisition

All images were acquired using the same microscopy settings within an experiment set (excitation light power, exposure time and number of *z*-stack) with its own untreated control. Confocal images were acquired from inverted confocal microscopes (LSM 710 and LSM 780 confocal microscope, Zeiss; VOX spinning-disk confocal microscope, Perkin Elmer) and a wide-field deconvolution microscope (GE Healthcare, DeltaVisionElite) with 40× or 63× oil objective and photomultiplier tube (PMT) detectors for differential interference contrast (DIC) images. For fixed immunofluorescence samples, *z*-stacks were captured with *z*-step interval of 0.5 μm. Live-cell imaging was acquired at 30-second intervals with a *z*-step interval of 1 μm. For chromatin fiber assays, images were acquired with a Nikon wide-field fluorescence microscope (Nikon 80i) with 40×0.75 NA air objective and the Nikon image acquisition software. The TIF files were then processed and quantified in the ImageJ platform (https://imagej.net/ij/).

In cases where data were taken using two different microscopes due to unavailability of the previous one, to account for this variation, we also imaged the control side by side for calculating the fold change of the intensity relative to its untreated control. Images within each panel are displayed using consistent contrast settings.

### Quantification of metaphase plate signal intensity

Metaphase fluorescence intensity was quantified as in Lee et al. (2016). Metaphase was judged as the time point right before noticeable separation of chromatin masses. The metaphase plate intensity was quantified by fitting a rectangle (A1) around the metaphase chromatin in a maximum-projected image (Fig. S2A). The area of A1 was kept constant within each set of experiments. Area 2 (A2) was a slightly bigger rectangle that enclosed A1. The average background intensity was calculated as follows:

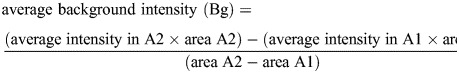


The signal intensity was then calculated as follows:

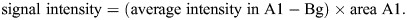


The signal intensity was then normalized to the untreated control to show the fold change. This normalization allowed us to display the metaphase plate intensity as a ratio or fold change relative to the untreated control.

### Profiling of prometaphase chromosome transverse intensity by line scan

Prometaphase chromosome line scan was performed to obtain a profile of signal intensity along the length of the straight line drawn. A line set with a width of 10 pixels was drawn along the lateral axis of prometaphase chromosomes. The average channel intensity of the 10 pixels was plotted against length using the ‘plot profile’ function in ImageJ. Different line scans were aligned to midpoints between the two peaks in the plot before composing the average line scans shown in Fig. 2B. Our inclusion criteria of an analyzable chromosome were: choosing cells with condensed chromosomes in prometaphase; choosing chromosomes that lie flat (the two centromeres on the *xy* plane instead of rotated in the *xz* plane or *yz* plane).

### Aneuploidy assay

The aneuploidy assay was performed as described in Lee et al. (2016). The degree of aneuploidy was assessed using the chromosome number reporter strain AV221. The strain carries *lacO* repeats inserted in the translocated segment of chromosome II/III and expresses LacI::GFP. Normal diploid embryonic cells are expected to have two GFP foci, which are from the homologous chromosomes. GFP::LacI was stained using anti-GFP antibodies (Table S3), and foci per embryonic cell was counted from the confocal images.

### Chromatin fiber preparation and analysis of foci

The preparation of chromatin fibers was adopted from Kyriacou and Heun (2018) (and personal communication with Eftychia Kyriacou). Chitinase-treated embryos were lysed and spread on glass slides until they reached a semi-dry state. Slides were incubated in lysis buffer (8 M urea and 200 mM NaCl) and pulled out slowly. The rigorous lysis condition (8 M urea) ensures removal of weakly interacting proteins but not nucleosomal proteins. Control and RNAi samples were prepared in parallel to ensure that a similar stretching force was applied. Our inclusion criteria for fibers: (1) fibers with noticeable GFP::HCP-3, histone H4 and DAPI staining were imaged; (2) fibers with similar chromatin width (determined from DAPI staining) were selected, and fiber areas were selected as 20-pixel-wide rectangles covering only the linear fibers for analysis. A threshold was set to distinguish the GFP foci signals from background intensity by autothresholding in ImageJ or by manually setting the threshold to 38 (arbitrary units, a.u.; Fig. S2C). A region of interest (ROI) was defined using the ‘Analyze particles’ function in ImageJ (circularity, 0.01–1.00; exclude particles on the edges). The area and the average intensities of GFP::HCP-3 and histone H4 staining of the foci ROI were quantified. To account for the possible variations in the degree of chromatin stretching, the GFP foci intensities were measured as a relative ratio between GFP::HCP-3 and histone H4. The nearest neighbor distance (NND) of the ROIs was calculated using the ‘NND’ function in ImageJ (Fig. 3E). NND represents the shortest distance for a given foci to its neighboring foci, indicating how densely foci are distributed. The NND was normalized by multiplying to H4 signal intensity for any given focus: 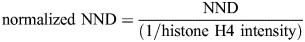, as more-compacted chromatin is expected to give a smaller NND and a higher H4 signal (Fig. S2D).

### Chromatin HCP-3 turnover photobleaching assay

The photobleaching assay was performed as described in Gassmann et al. (2012). Either untreated or *csr-1*(RNAi) OD421 (GFP::HCP-3; mCherry::H2B) hermaphrodites were dissected for live imaging using a VOX spinning-disk confocal microscope (Perkin Elmer). One-cell embryos were tracked until they reached the first anaphase. At anaphase, GFP on the posterior (P-side) sister was photobleached with the 440 nm laser (20%, 500 ms, 100 times). The photobleached embryo was imaged at 30–60 s intervals until the metaphases of the two-cell stage. The HCP-3 turnover within one cell division was calculated by the normalized GFP to mCherry intensity ratio of bleached P1 sister chromatid (B) over that of the unbleached AB sister chromatid (U). For control embryos that were not photobleached, the GFP to mCherry ratio of the P1 cell over the AB cell was calculated.

### RT-qPCR

Equal amounts of untreated or RNAi-treated worms (RNAi by injection) or embryos (RNAi by feeding) were harvested for RNA extraction using a standard TRIzol (Life Technologies) protocol. Reverse transcription was performed using reverse transcriptase (Applied Biosystems, 4368814). Real-time qPCR was performed in a StepOne Real-Time PCR System using SYBR green master mix (Applied Biosystems). Primers used for RT-qPCR are listed in Table S2.

### Western blotting

Embryos were lysed in 100 μl RIPA buffer (Millipore) using water bath sonication at 4°C for 30 min. Protein concentrations were determined by BCA assay (Pierce BCA Protein Assay Kit, Thermo Fisher Scientific). Then, 20–30 μg protein was loaded in each lane for SDS-PAGE. Proteins were transferred to PVDF membranes and blocked with 5% milk in Tris-buffered saline with 0.1% Triton X-100 (TBST) then probed with primary antibodies (Table S3) diluted in 5% milk in TBST at 4°C overnight. After washes with TBST, immunoblots were subjected to incubation with HRP-conjugated secondary antibodies (Abcam, ab97051 or ab97023) at room temperature for 1 h followed by washing. Blot signals were developed and detected using Amersham ECL Prime western blotting detection reagent (GE Healthcare Life Sciences). Full blots of the three independent biological replicates can be found in Fig. S4A.

### Statistics

*P*-values were calculated by two-tailed unpaired *t*-test (or Fisher's test for frequencies). Significance was reported if *P*<0.05. Error bar definitions are indicated in figure legends, representing s.d. or 95% confidence interval (signal intensity and chromatin fiber quantification) of the mean. ns, not significant; */^#^*P*<0.05; **/^##^*P*<0.01; ***/^###^*P*<0.001, ****/^####^*P*<0.0001. Graphs and plots were prepared with Microsoft Excel and Graphpad Prism.

## Supplementary Material



10.1242/joces.261895_sup1 Supplementary information

## References

[JCS261895C1] Amato, A., Schillaci, T., Lentini, L. and Di Leonardo, A. (2009). CENPA overexpression promotes genome instability in pRb-depleted human cells. *Mol. Cancer* 8, 119. 10.1186/1476-4598-8-119 20003272 PMC2797498

[JCS261895C2] Amor, D. J. and Choo, K. A. (2002). Neocentromeres: role in human disease, evolution, and centromere study. *Am. J. Hum. Genet.* 71, 695-714. 10.1086/342730 12196915 PMC378529

[JCS261895C3] Aoki, K., Moriguchi, H., Yoshioka, T., Okawa, K. and Tabara, H. (2007). In vitro analyses of the production and activity of secondary small interfering RNAs in C. elegans. *EMBO J.* 26, 5007-5019. 10.1038/sj.emboj.7601910 18007599 PMC2140100

[JCS261895C4] Avgousti, D. C., Palani, S., Sherman, Y. and Grishok, A. (2012). CSR–1 RNAi pathway positively regulates histone expression in C. elegans. *EMBO J.* 31, 3821-3832. 10.1038/emboj.2012.216 22863779 PMC3463841

[JCS261895C5] Batista, P. J., Ruby, J. G., Claycomb, J. M., Chiang, R., Fahlgren, N., Kasschau, K. D., Chaves, D. A., Gu, W., Vasale, J. J., Duan, S. et al. (2008). PRG-1 and 21U-RNAs interact to form the piRNA complex required for fertility in C. elegans. *Mol. Cell* 31, 67-78. 10.1016/j.molcel.2008.06.002 18571452 PMC2570341

[JCS261895C6] Billi, A. C., Fischer, S. E. and Kim, J. K. (2018). Endogenous RNAi pathways in C. elegans. In: *WormBook* (ed. The *C. elegans* Research Community). Wormbook. 10.1895/wormbook.1.170.1 PMC4781133 24816713

[JCS261895C7] Buchwitz, B. J., Ahmad, K., Moore, L. L., Roth, M. B. and Henikoff, S. (1999). A histone-H3-like protein in C. elegans. *Nature* 401, 547-548. 10.1038/44062 10524621

[JCS261895C9] Castillo, A. G., Mellone, B. G., Partridge, J. F., Richardson, W., Hamilton, G. L., Allshire, R. C. and Pidoux, A. L. (2007). Plasticity of fission yeast CENP-A chromatin driven by relative levels of histone H3 and H4. *PLoS Genet.* 3, e121. 10.1371/journal.pgen.0030121 17677001 PMC1934396

[JCS261895C10] Cecere, G., Hoersch, S., O'keeffe, S., Sachidanandam, R. and Grishok, A. (2014). Global effects of the CSR-1 RNA interference pathway on the transcriptional landscape. *Nat. Struct. Mol. Biol.* 21, 358-365. 10.1038/nsmb.2801 24681887 PMC4068146

[JCS261895C11] Charlesworth, A. G., Seroussi, U., Lehrbach, N., Renaud, M. S., Sundby, A. E., Molnar, R. I., Lao, R. X., Willis, A. R., Woock, J. R., Aber, M. J. et al. (2021). Two isoforms of the essential C. elegans Argonaute CSR-1 differentially regulate sperm and oocyte fertility. *Nucleic Acids Res.* 49, 8836-8865. 10.1093/nar/gkab619 34329465 PMC8421154

[JCS261895C12] Cimini, D. (2007). Detection and correction of merotelic kinetochore orientation by Aurora B and its partners. *Cell Cycle* 6, 1558-1564. 10.4161/cc.6.13.4452 17603301

[JCS261895C13] Claycomb, J. M., Batista, P. J., Pang, K. M., Gu, W., Vasale, J. J., Van Wolfswinkel, J. C., Chaves, D. A., Shirayama, M., Mitani, S., Ketting, R. F. et al. (2009). The Argonaute CSR-1 and its 22G-RNA cofactors are required for holocentric chromosome segregation. *Cell* 139, 123-134. 10.1016/j.cell.2009.09.014 19804758 PMC2766185

[JCS261895C14] De Rop, V., Padeganeh, A. and Maddox, P. S. (2012). CENP-A: the key player behind centromere identity, propagation, and kinetochore assembly. *Chromosoma* 121, 527-538. 10.1007/s00412-012-0386-5 23095988 PMC3501172

[JCS261895C15] Espeut, J., Cheerambathur, D. K., Krenning, L., Oegema, K. and Desai, A. (2012). Microtubule binding by KNL-1 contributes to spindle checkpoint silencing at the kinetochore. *J. Cell Biol.* 196, 469-482. 10.1083/jcb.201111107 22331849 PMC3284002

[JCS261895C16] Essex, A., Dammermann, A., Lewellyn, L., Oegema, K. and Desai, A. (2009). Systematic analysis in Caenorhabditis elegans reveals that the spindle checkpoint is composed of two largely independent branches. *Mol. Biol. Cell* 20, 1252-1267. 10.1091/mbc.e08-10-1047 19109417 PMC2642744

[JCS261895C17] Eulalio, A., Huntzinger, E. and Izaurralde, E. (2008). GW182 interaction with Argonaute is essential for miRNA-mediated translational repression and mRNA decay. *Nat. Struct. Mol. Biol.* 15, 346. 10.1038/nsmb.1405 18345015

[JCS261895C18] Foltz, D. R., Jansen, L. E., Bailey, A. O., Yates, J. R., Bassett, E. A., Wood, S., Black, B. E. and Cleveland, D. W. (2009). Centromere-specific assembly of CENP-a nucleosomes is mediated by HJURP. *Cell* 137, 472-484. 10.1016/j.cell.2009.02.039 19410544 PMC2747366

[JCS261895C19] Friend, K., Campbell, Z. T., Cooke, A., Kroll-Conner, P., Wickens, M. P. and Kimble, J. (2012). A conserved PUF–Ago–eEF1A complex attenuates translation elongation. *Nat. Struct. Mol. Biol.* 19, 176. 10.1038/nsmb.2214 22231398 PMC3293257

[JCS261895C20] Fukagawa, T. and Earnshaw, W. C. (2014). The centromere: chromatin foundation for the kinetochore machinery. *Dev. Cell* 30, 496-508. 10.1016/j.devcel.2014.08.016 25203206 PMC4160344

[JCS261895C21] Gassmann, R., Rechtsteiner, A., Yuen, K. W., Muroyama, A., Egelhofer, T., Gaydos, L., Barron, F., Maddox, P., Essex, A., Monen, J. et al. (2012). An inverse relationship to germline transcription defines centromeric chromatin in C. elegans. *Nature* 484, 534-537. 10.1038/nature10973 22495302 PMC3538161

[JCS261895C22] Gerson-Gurwitz, A., Wang, S., Sathe, S., Green, R., Yeo, G. W., Oegema, K. and Desai, A. (2016). A small RNA-catalytic Argonaute pathway tunes germline transcript levels to ensure embryonic divisions. *Cell* 165, 396-409. 10.1016/j.cell.2016.02.040 27020753 PMC4826293

[JCS261895C23] Gregan, J., Polakova, S., Zhang, L., Tolić-Nørrelykke, I. M. and Cimini, D. (2011). Merotelic kinetochore attachment: causes and effects. *Trends Cell Biol.* 21, 374-381. 10.1016/j.tcb.2011.01.003 21306900 PMC3117139

[JCS261895C24] Gu, W., Shirayama, M., Conte, D., Vasale, J., Batista, P. J., Claycomb, J. M., Moresco, J. J., Youngman, E. M., Keys, J. and Stoltz, M. J. et al. (2009). Distinct argonaute-mediated 22G-RNA pathways direct genome surveillance in the C. elegans germline. *Mol. Cell* 36, 231-244. 10.1016/j.molcel.2009.09.020 19800275 PMC2776052

[JCS261895C25] Heun, P., Erhardt, S., Blower, M. D., Weiss, S., Skora, A. D. and Karpen, G. H. (2006). Mislocalization of the Drosophila centromere-specific histone CID promotes formation of functional ectopic kinetochores. *Dev. Cell* 10, 303-315. 10.1016/j.devcel.2006.01.014 16516834 PMC3192491

[JCS261895C26] Huang, C., Wang, X., Liu, X., Cao, S. and Shan, G. (2015). RNAi pathway participates in chromosome segregation in mammalian cells. *Cell Discov.* 1, 15029. 10.1038/celldisc.2015.29 27462427 PMC4860838

[JCS261895C27] Jansen, L. E., Black, B. E., Foltz, D. R. and Cleveland, D. W. (2007). Propagation of centromeric chromatin requires exit from mitosis. *J. Cell Biol.* 176, 795-805. 10.1083/jcb.200701066 17339380 PMC2064054

[JCS261895C28] Kaitna, S., Pasierbek, P., Jantsch, M., Loidl, J. and Glotzer, M. (2002). The aurora B kinase AIR-2 regulates kinetochores during mitosis and is required for separation of homologous chromosomes during meiosis. *Curr. Biol.* 12, 798-812. 10.1016/S0960-9822(02)00820-5 12015116

[JCS261895C29] Kapoor, T. M. (2004). Chromosome segregation: correcting improper attachment. *Curr. Biol.* 14, R1011-R1013. 10.1016/j.cub.2004.11.026 15589138

[JCS261895C30] Kyriacou, E. and Heun, P. (2018). High-resolution mapping of centromeric protein association using APEX-chromatin fibers. *Epigenetics Chromatin* 11, 68. 10.1186/s13072-018-0237-6 30445992 PMC6238281

[JCS261895C31] Ladouceur, A.-M., Ranjan, R., Smith, L., Fadero, T., Heppert, J., Goldstein, B., Maddox, A. S. and Maddox, P. S. (2017). CENP-A and topoisomerase-II antagonistically affect chromosome length. *J. Cell Biol.* 216, 2645-2655. 10.1083/jcb.201608084 28733327 PMC5584148

[JCS261895C32] Lee, H.-C., Gu, W., Shirayama, M., Youngman, E., Conte, D. and Mello, C. C. (2012). C. elegans piRNAs mediate the genome-wide surveillance of germline transcripts. *Cell* 150, 78-87. 10.1016/j.cell.2012.06.016 22738724 PMC3410639

[JCS261895C33] Lee, B. C. H., Lin, Z. and Yuen, K. W. Y. (2016). RbAp46/48 LIN-53 Is Required for Holocentromere Assembly in Caenorhabditis elegans. *Cell Rep.* 14, 1819-1828. 10.1016/j.celrep.2016.01.065 26904949

[JCS261895C34] Levesque, A. A. and Compton, D. A. (2001). The chromokinesin Kid is necessary for chromosome arm orientation and oscillation, but not congression, on mitotic spindles. *J. Cell Biol.* 154, 1135-1146. 10.1083/jcb.200106093 11564754 PMC2150818

[JCS261895C62] Lin, Z. and Yuen, K. W. Y. (2021). RbAp46/48LIN-53 and HAT-1 are required for initial CENP-AHCP-3 deposition and de novo holocentromere formation on artificial chromosomes in *Caenorhabditis elegans* embryos. *Nucleic Acids Res.* 49, 9154-9173. 10.1093/nar/gkab217 33872374 PMC8450102

[JCS261895C35] Lin, Z., Xie, Y., Nong, W., Ren, X., Li, R., Zhao, Z., Hui, J. H. L. and Yuen, K. W. Y. (2021). Formation of artificial chromosomes in Caenorhabditis elegans and analyses of their segregation in mitosis, DNA sequence composition and holocentromere organization. *Nucleic Acids Res.* 49, 9174-9193. 10.1093/nar/gkab690 34417622 PMC8450109

[JCS261895C36] Liu, J., Carmell, M. A., Rivas, F. V., Marsden, C. G., Thomson, J. M., Song, J.-J., Hammond, S. M., Joshua-Tor, L. and Hannon, G. J. (2004). Argonaute2 is the catalytic engine of mammalian RNAi. *Science* 305, 1437-1441. 10.1126/science.1102513 15284456

[JCS261895C37] Liu, J., Rivas, F. V., Wohlschlegel, J., Yates, J. R., Parker, R. and Hannon, G. J. (2005). A role for the P-body component GW182 in microRNA function. *Nat. Cell Biol.* 7, 1261-1266. 10.1038/ncb1333 16284623 PMC1804202

[JCS261895C38] Maddox, P. S., Hyndman, F., Monen, J., Oegema, K. and Desai, A. (2007). Functional genomics identifies a Myb domain–containing protein family required for assembly of CENP-A chromatin. *J. Cell Biol.* 176, 757-763. 10.1083/jcb.200701065 17339379 PMC2064049

[JCS261895C39] Maiato, H., Gomes, A. M., Sousa, F. and Barisic, M. (2017). Mechanisms of chromosome congression during mitosis. *Biology* 6, 13. 10.3390/biology6010013 28218637 PMC5372006

[JCS261895C40] Mcewen, B. F., Hsieh, C. E., Mattheyses, A. L. and Rieder, C. L. (1998). A new look at kinetochore structure in vertebrate somatic cells using high-pressure freezing and freeze substitution. *Chromosoma* 107, 366-375. 10.1007/s004120050320 9914368 PMC2905855

[JCS261895C41] Mckinley, K. L. and Cheeseman, I. M. (2016). The molecular basis for centromere identity and function. *Nat. Rev. Mol. Cell Biol.* 17, 16-29. 10.1038/nrm.2015.5 26601620 PMC8603311

[JCS261895C42] Mellone, B. G., Grive, K. J., Shteyn, V., Bowers, S. R., Oderberg, I. and Karpen, G. H. (2011). Assembly of Drosophila centromeric chromatin proteins during mitosis. *PLoS Genet.* 7, e1002068. 10.1371/journal.pgen.1002068 21589899 PMC3093364

[JCS261895C43] Montgomery, B. E., Vijayasarathy, T., Marks, T. N., Cialek, C. A., Reed, K. J. and Montgomery, T. A. (2021). Dual roles for piRNAs in promoting and preventing gene silencing in C. elegans. *Cell Rep.* 37, 110101. 10.1016/j.celrep.2021.110101 34879267 PMC8730336

[JCS261895C44] Nguyen, D. A. H. and Phillips, C. M. (2021). Arginine methylation promotes siRNA-binding specificity for a spermatogenesis-specific isoform of the Argonaute protein CSR-1. *Nat. Commun.* 12, 4212. 10.1038/s41467-021-24526-6 34244496 PMC8270938

[JCS261895C63] O'Connell, K. F., Caron, C., Kopish, K. R., Hurd, D. D., Kemphues, K. J., Li, Y. and White, J. G. (2001). The *C. elegans* zyg-1 gene encodes a regulator of centrosome duplication with distinct maternal and paternal roles in the embryo. *Cell* 105, 547-558. 10.1016/s0092-8674(01)00338-5 11371350

[JCS261895C45] Oegema, K., Desai, A., Rybina, S., Kirkham, M. and Hyman, A. A. (2001). Functional analysis of kinetochore assembly in Caenorhabditis elegans. *J. Cell Biol.* 153, 1209-1226. 10.1083/jcb.153.6.1209 11402065 PMC2192036

[JCS261895C46] Powers, J., Rose, D. J., Saunders, A., Dunkelbarger, S., Strome, S. and Saxton, W. M. (2004). Loss of KLP-19 polar ejection force causes misorientation and missegregation of holocentric chromosomes. *J. Cell Biol.* 166, 991-1001. 10.1083/jcb.200403036 15452142 PMC1534123

[JCS261895C47] Quarato, P., Singh, M., Cornes, E., Li, B., Bourdon, L., Mueller, F., Didier, C. and Cecere, G. (2021). Germline inherited small RNAs facilitate the clearance of untranslated maternal mRNAs in C. elegans embryos. *Nat. Commun.* 12, 1441. 10.1038/s41467-021-21691-6 33664268 PMC7933186

[JCS261895C48] Redemann, S., Baumgart, J., Lindow, N., Shelley, M., Nazockdast, E., Kratz, A., Prohaska, S., Brugués, J., Fürthauer, S. and Müller-Reichert, T. (2017). C. elegans chromosomes connect to centrosomes by anchoring into the spindle network. *Nat. Commun.* 8, 15288. 10.1038/ncomms15288 28492281 PMC5437269

[JCS261895C49] Schwarz, D. S., Tomari, Y. and Zamore, P. D. (2004). The RNA-induced silencing complex is a Mg2+-dependent endonuclease. *Curr. Biol.* 14, 787-791. 10.1016/j.cub.2004.03.008 15120070

[JCS261895C50] Scott, K. C., Merrett, S. L. and Willard, H. F. (2006). A heterochromatin barrier partitions the fission yeast centromere into discrete chromatin domains. *Curr. Biol.* 16, 119-129. 10.1016/j.cub.2005.11.065 16431364

[JCS261895C51] Seth, M., Shirayama, M., Gu, W., Ishidate, T., Conte, D. and Mello, C. C. (2013). The C. elegans CSR-1 argonaute pathway counteracts epigenetic silencing to promote germline gene expression. *Dev. Cell* 27, 656-663. 10.1016/j.devcel.2013.11.014 24360782 PMC3954781

[JCS261895C52] Singh, M., Cornes, E., Li, B., Quarato, P., Bourdon, L., Dingli, F., Loew, D., Proccacia, S. and Cecere, G. (2021). Translation and codon usage regulate Argonaute slicer activity to trigger small RNA biogenesis. *Nat. Commun.* 12, 3492. 34108460 10.1038/s41467-021-23615-w PMC8190271

[JCS261895C53] Tanaka, T. U. (2002). Bi-orienting chromosomes on the mitotic spindle. *Curr. Opin. Cell Biol.* 14, 365-371. 10.1016/S0955-0674(02)00328-9 12067660

[JCS261895C54] Tomonaga, T., Matsushita, K., Yamaguchi, S., Oohashi, T., Shimada, H., Ochiai, T., Yoda, K. and Nomura, F. (2003). Overexpression and mistargeting of centromere protein-A in human primary colorectal cancer. *Cancer Res.* 63, 3511-3516. 12839935

[JCS261895C55] Tu, S., Wu, M. Z., Wang, J., Cutter, A. D., Weng, Z. and Claycomb, J. M. (2015). Comparative functional characterization of the CSR-1 22G-RNA pathway in Caenorhabditis nematodes. *Nucleic Acids Res.* 43, 208-224. 10.1093/nar/gku1308 25510497 PMC4288196

[JCS261895C56] Van Wolfswinkel, J. C., Claycomb, J. M., Batista, P. J., Mello, C. C., Berezikov, E. and Ketting, R. F. (2009). CDE-1 affects chromosome segregation through uridylation of CSR-1-bound siRNAs. *Cell* 139, 135-148. 10.1016/j.cell.2009.09.012 19804759

[JCS261895C57] Wedeles, C. J., Wu, M. Z. and Claycomb, J. M. (2013a). A multitasking Argonaute: exploring the many facets of C. elegans CSR-1. *Chromosome Res.* 21, 573-586. 10.1007/s10577-013-9383-7 24178449

[JCS261895C58] Wedeles, C. J., Wu, M. Z. and Claycomb, J. M. (2013b). Protection of germline gene expression by the C. elegans Argonaute CSR-1. *Dev. Cell* 27, 664-671. 10.1016/j.devcel.2013.11.016 24360783

[JCS261895C59] Xu, F., Feng, X., Chen, X., Weng, C., Yan, Q., Xu, T., Hong, M. and Guang, S. (2018). A cytoplasmic Argonaute protein promotes the inheritance of RNAi. *Cell Rep.* 23, 2482-2494. 10.1016/j.celrep.2018.04.072 29791857

[JCS261895C60] Yigit, E., Batista, P. J., Bei, Y., Pang, K. M., Chen, C.-C. G., Tolia, N. H., Joshua-Tor, L., Mitani, S., Simard, M. J. and Mello, C. C. (2006). Analysis of the C. elegans Argonaute family reveals that distinct Argonautes act sequentially during RNAi. *Cell* 127, 747-757. 10.1016/j.cell.2006.09.033 17110334

[JCS261895C61] Youngman, E. M. and Claycomb, J. M. (2014). From early lessons to new frontiers: the worm as a treasure trove of small RNA biology. *Front. Genet.* 5, 416. 10.3389/fgene.2014.00416 25505902 PMC4245922

