# Supplementary material for: Argonaute protein CSR-1 restricts localization of holocentromere protein HCP-3, the C. elegans CENP-A homolog
Source: J Cell Sci. 2024 Sep 18;137(18):jcs261895. doi: 10.1242/jcs.261895 (PMC11423810; doi:10.1242/jcs.261895)
Supplement: Supplementary information [file joces-137-261895-s1.pdf]

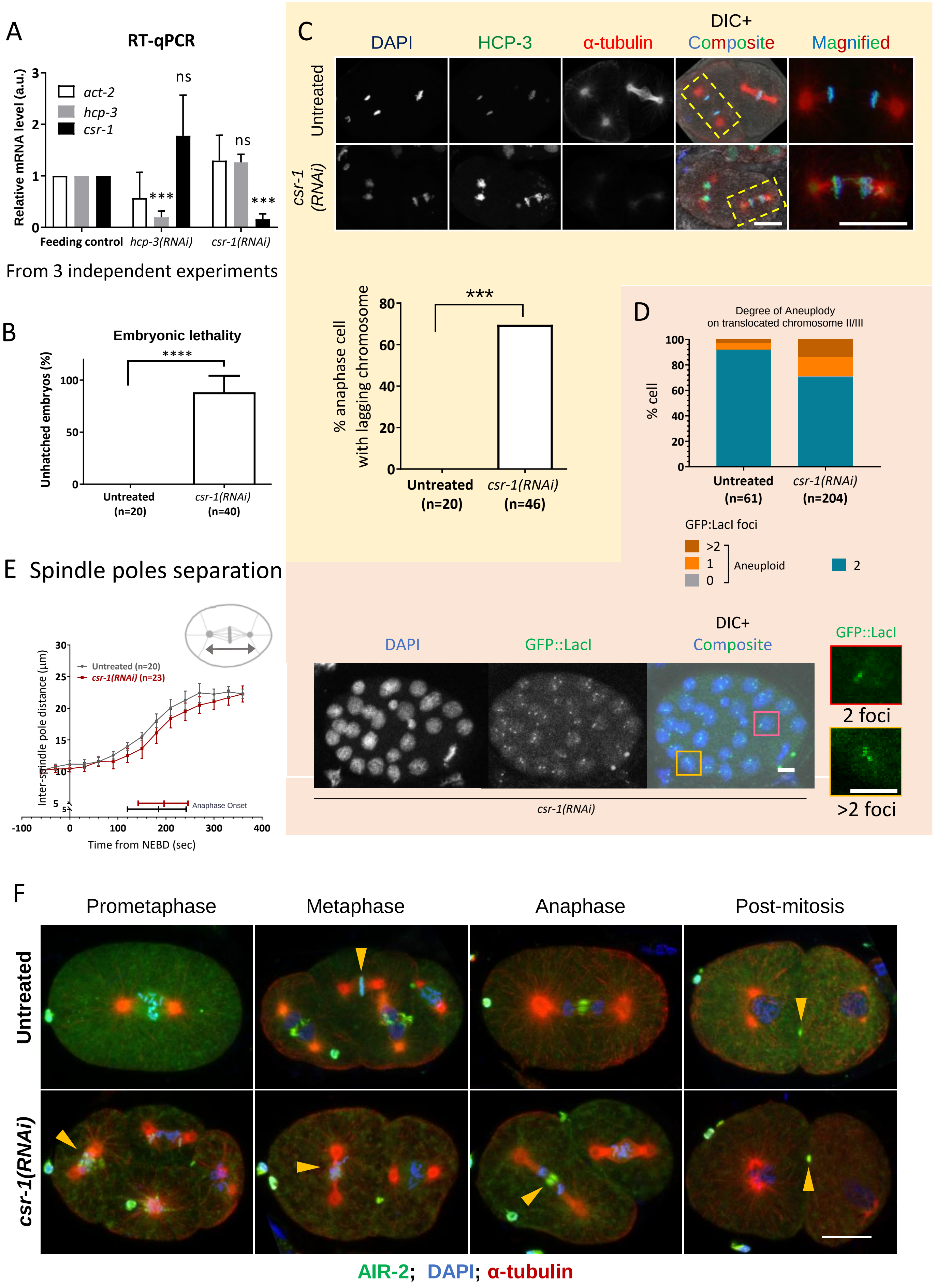

Journal of Cell Science • Supplementary information

Fig. S1. Verification of *csr-1* RNA knockdown efficiency and its effect on AIR-2 localization.

(A) *hcp-3* and *csr-1* RNA level with *hcp-3* or *csr-1*(RNAi) knockdown. *act-2* was used as the loading control. Three independent biological replicates of each treatment were used. Error bars represent the SD of the mean. *: p < 0.05, ***: p < 0.001, ns: not significant, unpaired t-test.

(B) Percentage of embryonic lethality of the untreated and *csr-1*(RNAi) knockdown worms. n equals the number of untreated or RNAi-treated animals used. Error bars represent the SD of the mean. ****: p < 0.001, unpaired t-test.

(C) Untreated control or *csr-1*(RNAi) embryos are immunostained for HCP-3 and α-tubulin with DAPI-staining. Examples of a normal versus a missegregated anaphase. Chromosomes are highlighted in dashed rectangles and enlarged on the right. Scale bars: 10 μm. The percentage of lagging chromosomes observed was quantified. n equals the number of anaphase cells analyzed. ***: p < 0.001, Fisher's exact test.

(D) Embryos containing GFP::LacI-labelled LacO repeats on the translocated Chromosome II/III were immunostained for GFP with DAPI staining, showing the copy number of the translocated chromosome. Examples of a normal cell versus an aneuploid cell are highlighted in dashed rectangles and enlarged on the right. Scale bars: 5 μm. The percentage of aneuploid cells was scored in the untreated and *csr-1*(RNAi) embryos. n equals the number of cells analyzed. ***: p < 0.001, Fisher's exact test.

(E) The distance between spindle poles in mitosis in one-cell embryos was measured in different RNAi treatments. untreated control and *csr-1*(RNAi) embryos. n equals the number of embryos analyzed. The timing for anaphase onset for untreated and *csr-1*(RNAi) P0 embryos were extracted from **Fig. 1B**. Error bars represent the SD of the mean. *: p < 0.05; **: p < 0.01; ***: p < 0.001; ns: not significant, unpaired t-test.

(F) Immunofluorescence image showing AIR-2 localization in early embryos at different mitotic stages. Scale bar: 10 μm.

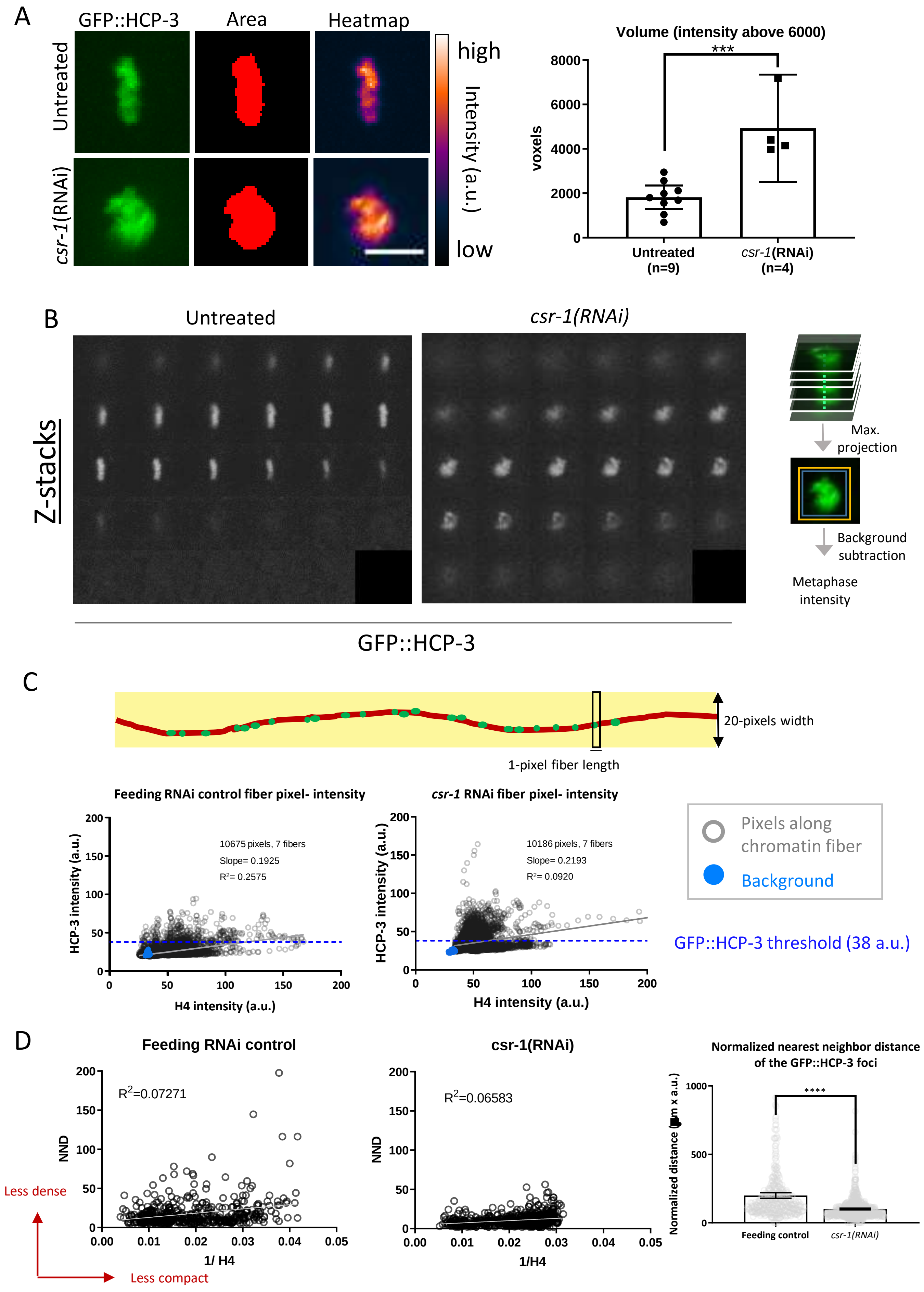

Journal of Cell Science • Supplementary information

Fig. S2. Supporting experiment details to decipher the effect of *csr-1*(RNAi) on HCP-3.

(A) Metaphase chromatin in the *csr-1*(RNAi) embryos show an increase in area (and volume) and an increase in the number of high-intensity GFP::HCP-3 pixels (orange). The "Area" shown are the ROIs with GFP intensity > 6000 a.u. The heatmap shows the pixel intensity with LUT labelled on the right. Scale bar: 10 μm. Quantification of the volume of metaphase plate in untreated control and the *csr-1*(RNAi) embryos. Error bars represent 95% confidence interval of the means. ***: $p < 0.001$, ns: not significant, unpaired t-test. n equals number of one-cell embryos quantified.

(B) Z-stack images of the metaphase chromatin in untreated and *csr-1*(RNAi) embryos. Illustration showing how the metaphase plate total intensities were quantified in this study (See also in materials and methods). First, Z-stack images were maximum projected. Around the chromatin, two rectangular ROIs with different sizes were defined. The area between the two rectangles were taken for calculating the background intensity. The mean background intensity was then subtracted from the smaller ROI measured mean intensity. This provided the mean metaphase chromatin intensity for the ROI that were plotted. The ROI selected for all samples remain the same.

(C) Scatter plots of the GFP::HCP-3 and H4 pixel intensity of the pixels on the chromatin fibers prepared from the control (7 fibers, 10675 pixels) and the *csr-1*(RNAi) embryos (7 fibers, 10186 pixels). Segment lines (20-pixel-wide) were drawn along the image of fibers. The mean intensity of these 20 pixels was determined for each pixel-length on the fiber. The linear regression lines and the R-square values were indicated. Background pixels (blue) were obtained by sampling at the non-fiber background (feeding RNAi control: 1558 pixels ; *csr-1*(RNAi): 1581 pixels). A threshold for GFP::HCP-3 intensity was selected (38 a.u., blue dashed lines) to exclude fiber pixels with HCP-3 intensity close to the background level for both the feeding control and the *csr-1*(RNAi) embryos.

(D) Scatter plots of the nearest neighboring distance in pixels (NND) and the inverse of H4 intensity of each defined ROI from the chromatin fibers. (Left) Feeding RNAi control (Right) *csr-1*(RNAi). The linear regression lines are shown in grey.

Journal of Cell Science • Supplementary information

Fig. S3. chromatin fiber with GFP::HCP-3 foci (Scale bars: 5 µm)

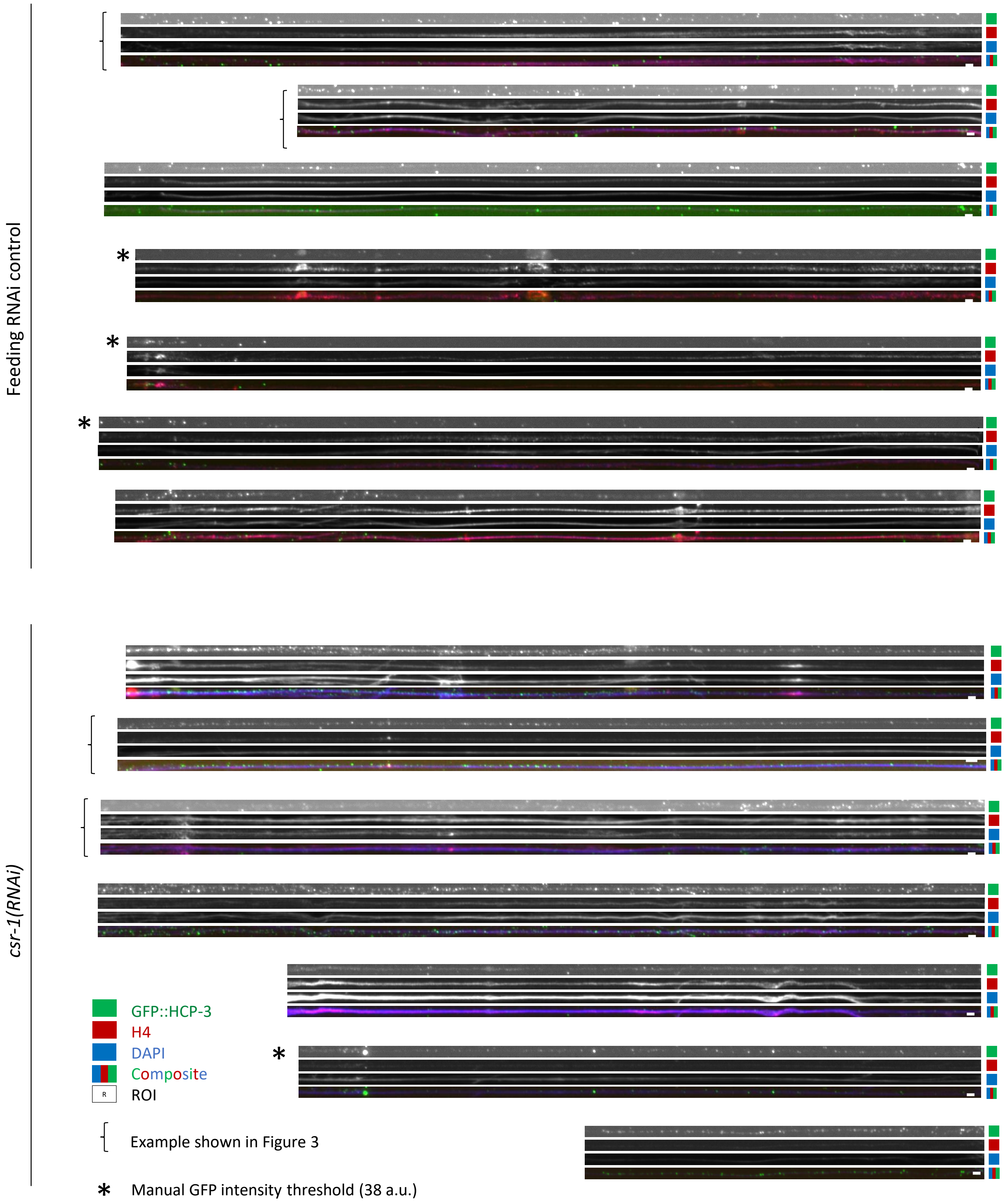

Journal of Cell Science • Supplementary information

Fig. S3. Collection of chromatin fiber with GFP::HCP-3 foci used in our analysis.

Chromatin were pulled from the lysed embryos on a glass slide. The slide with stretched chromatin were then fixed and stained with anti-GFP, anti-H4, and DAPI. The fiber with discrete GFP dot signal were chosen for further quantification for the properties. ROI were defined from binary image of GFP::HCP-3 channel after applying threshold and watershed. Particle ROI were defined based on their size and circularity. We compared their foci size, foci intensity, foci density (NND) difference between the csr-1 knockdown samples and the feeding RNAi control (Figure 3C-E). The 2 representative fiber images shown in Figure 3A are annotated with open bracket on the left.

Journal of Cell Science • Supplementary information

A

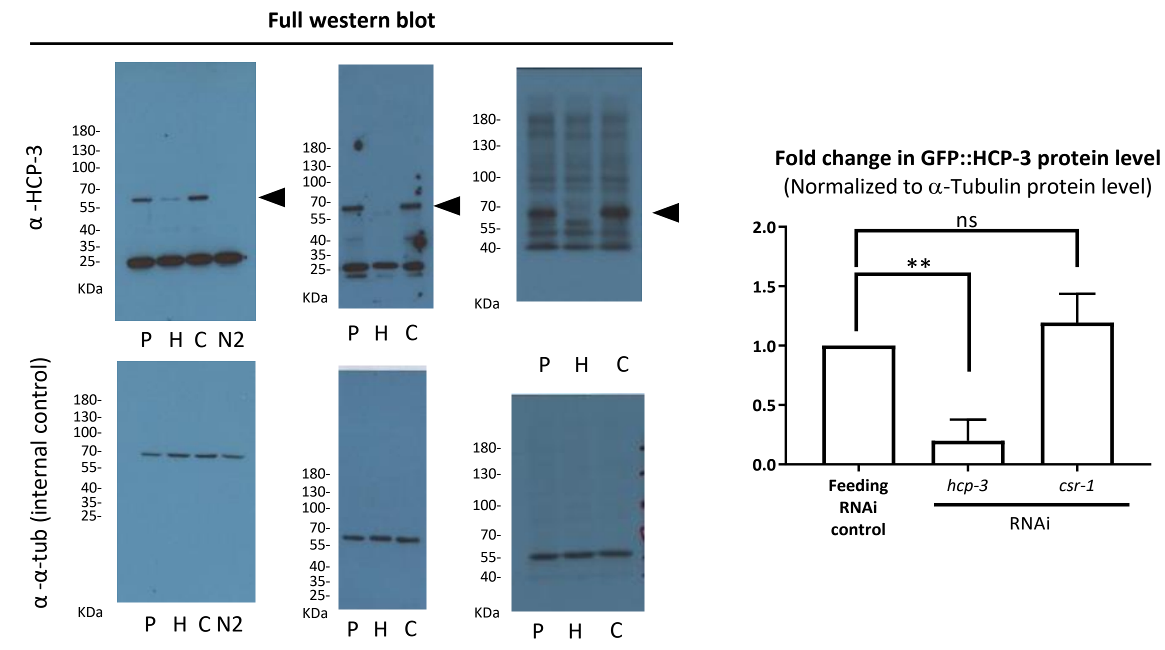

B

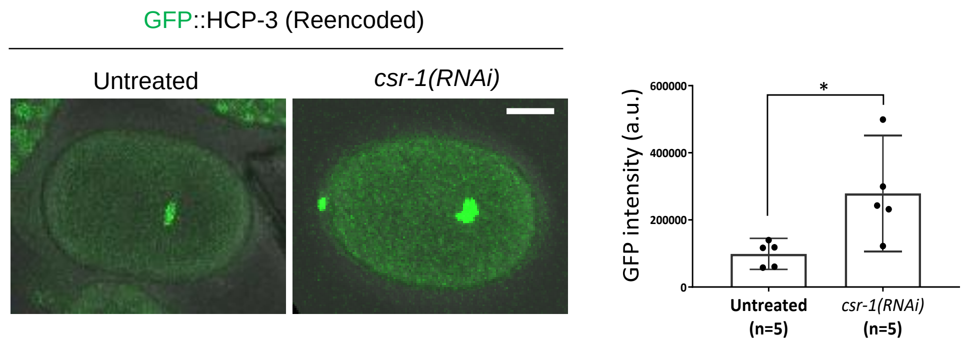

C

Gene	22G RNA CSR-1 IP Read count	Ratio of RNA level between *csr-1* hypomorph and N2*	Fold change of protein level (*csr-1* SIN;*csr-1(tm892)* to N2)
	(Claycomb et al., 2009)		(Gerson-gurwitz, et al., 2016)
hcp-3	1024.5	0.547	1-1.5
knl-2	588.5	0.449	1-1.5
knl-1	1145	0.531	1-1.5
hcp-4	1033	0.602	
mis-12	103	0.392	
ndc-80	327	0.425	
hcp-1	2631	0.436	2-2.5
knl-3	691.5	0.438	

Down-regulated 0.5 Up-regulated

*: Ratio is calculated as: wild type/(wild type+*csr-1(tm892)*) using the normalized intensity value.
If mRNA level is increased 2 fold in *csr-1(tm892)*: RATIO=1/3=0.33;
if it is reduced 2 fold: RATIO=1/1.5=0.66;
if no change: RATIO=1/2=0.5.
Red indicates genes that are upregulated in *csr-1(tm892)*, and blue indicates genes that are down-regulated in *csr-1(tm892).*

Journal of Cell Science • Supplementary information

Fig. S4. Supplementary data on how *csr-1(*RNAi) knockdown affects HCP-3 protein expression level, and the increase of HCP-3 on chromatin in *csr-1(*RNAi) occurs regardless of the reencoding of the hcp-3 gene.

(A) Full blots of the immunoblot in Figure 4B. Three biological replicates were performed. P: Feeding RNAi control (PL4440 empty vector); H: *hcp-3* feeding RNAi knockdown; C: *csr-1* feeding RNAi knockdown; N2: control strain without GFP fused HCP-3. The HCP-3 level of the RNAi-treated embryos is normalized to the feeding RNAi control, and then to the corresponding α-tubulin level. Error bars represent SD of the mean. **: $p < 0.01$, ns: not significant, unpaired t-test.

(B) Knockdown CSR-1 in strain with *hcp-3* gene reencoded. The strain WYY31 contains a reencoded *hcp-3* gene from OD1174 with endogenous *hcp-3* knockout allele from VC1393. Scale bar: 10 µm. The GFP intensity of the metaphase plate was quantified. n equals the number of embryos analyzed. Error bars represent 95% confidence interval of the mean. *: $p < 0.05$, unpaired t-test.

(C) Table summarizing published data of the number of complementary small RNAs and the expression in CSR-1-depleted or mutated condition of the genes that we tested in Figure 2. CSR-1 IP-small RNA-seq data is extracted from Claycomb et al., 2009 (From Supplementary mmc4), the transcriptome of wildtype and *csr-1* hypomorph (tm892) is extracted from Claycomb et al., 2009 (From Supplementary mmc6). The change in embryonic protein level comparing *csr-1^{SIN};csr-1(tm892)* worms to N2 *wild type* is extracted from Gerson-gurwitz et al., 2016.

Journal of Cell Science • Supplementary information

A

Embryonic lethality			
	RNAi	%	n
Injection	*csr-1 (0.5x)*	51.4	7
	lin-53 (0.5x)	100	3
	knl-2 (0.5x)	100	4
	lin-53 csr-1	100	3
	knl-2 csr-1	100	4
	lin-53 prg-1	93	3
	knl-2 prg-1	100	3
	csr-1 (0.5x)	46	3
	hrde-1	0	4
	hrde-1 csr-1	33	3
	prg-1 csr-1	100	2
	ego-1	100	3
	csr-1 (a&b)	91.2	2
	csr-1a	0	4
	csr-1 klp-7	100	3
	csr-1, with csr-1WT	95.2	2
	csr-1, with csr-1SIN	100	2

B

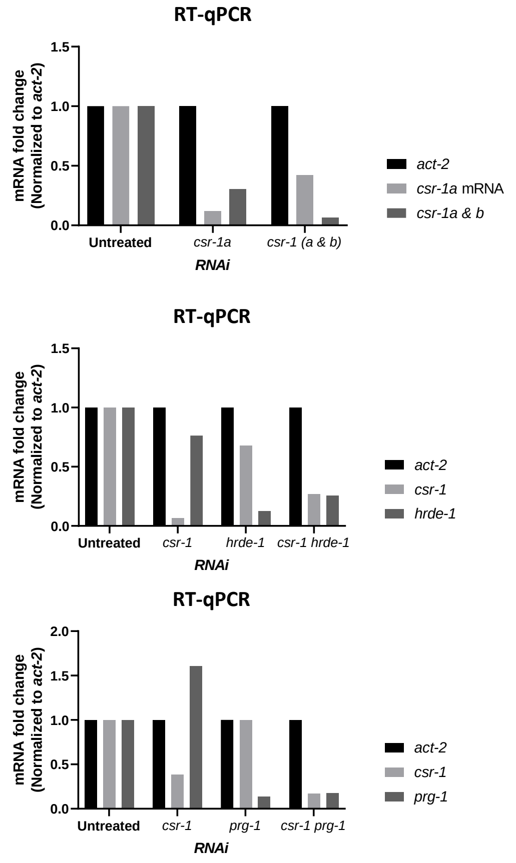

C

Misalignment & missegregation				
	RNAi	Misalign %	Misseg %	n
Injection	*csr-1 (a&b)*	100	100	6
	csr-1a	0	0	6
	csr-1 klp-7	40	80	5

D

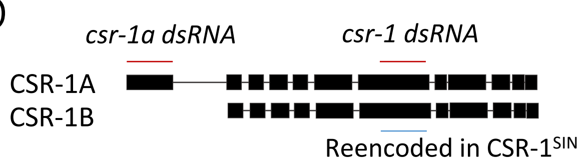

E

dsRNA conc. (μg/μl)	**csr-1**	**0**	←	**0.75**	→		**0**
	hcp-3	0.75	0.15	0.075	0.0075	0	0
	GFP::HCP-3; mCherry::H2B +DIC	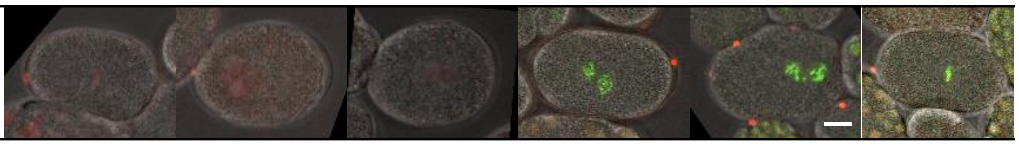					
Relative intensity to untreated control		0.06	0.06	0.04	2.13	2.33	1
Missegregation		Y	Y	Y	Y	Y	N
Embryonic lethality		79.5%	88.5%	100%	100%	61.8%	0%

F

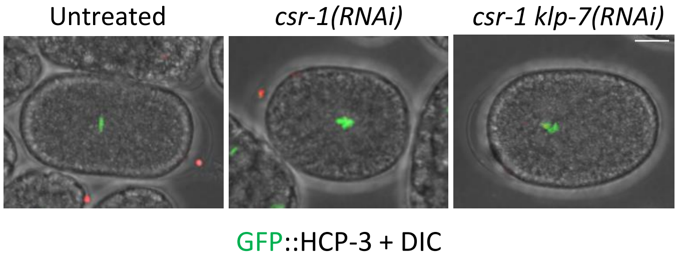

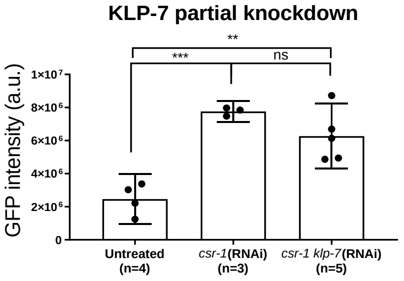

Journal of Cell Science • Supplementary information

Fig. S5. Verification of RNAi knockdown efficiency, transgene expression, segregation fate, and embryonic lethality

(A) Embryonic lethality of different RNAi treatments used in our study. Hermaphrodites were isolated 24 hours post-RNAi treatment on plates and let to lay embryos for 8-12 hours. The percentage of unhatched embryos for each hermaphrodite was quantified. The average percentage was shown and n equals number of hermaphrodites used.

(B) Quantification of RNA levels of *csr-1* isoforms, *hrde-1*, *prg-1* and *act-2* by RT-qPCR in *hrde-1*(RNAi), *prg-1*(RNAi) and *csr-1a*(RNAi) worms or double RNAi-treated worms. 2 technical qPCR replicates were performed, and the mean of the 2 replicates were plotted.

(C) Frequency of misalignment and missegregation in *csr-1 klp-7*(RNAi) knockdown embryos.

(D) Gene annotation of two *csr-1* isoforms. Positions for dsRNA design to knockdown *csr-1* and specifically knockdown *csr-1a* are in red. The region reencoded in the CSR-1 mutant (WT/SIN) is highlighted in blue. Note that the reencoded region overlaps with the *csr-1* dsRNA, so the mutants used in Figure 4 are RNAi-resistant. Other *csr-1* knockdown in this study use dsRNA targeting the exon 6-8 of the isoform B.

(E) HCP-3 partial knockdown in *csr-1(RNAi)* to observe if reducing HCP-3 level can rescue the chromosome missegregation phenotype. The ratio of chromatin GFP::HCP-3 and mCherry::H2B signals intensity relative to the untreated control, the fate of the chromosome segregation at anaphase, and the embryonic lethality are shown.

(F) Quantification of the mean GFP::HCP-3 intensity of the metaphase plate in one-cell embryos with partial knockdown of KLP-7 in *csr-1(RNAi)* embryos. Error bars represent 95% of the confidence interval. **: $p < 0.01$, ***: $p < 0.001$, ns: not significant, unpaired t-test.

Journal of Cell Science • Supplementary information

Fig. S6. Double RNAi knockdown control for Fig. 5.

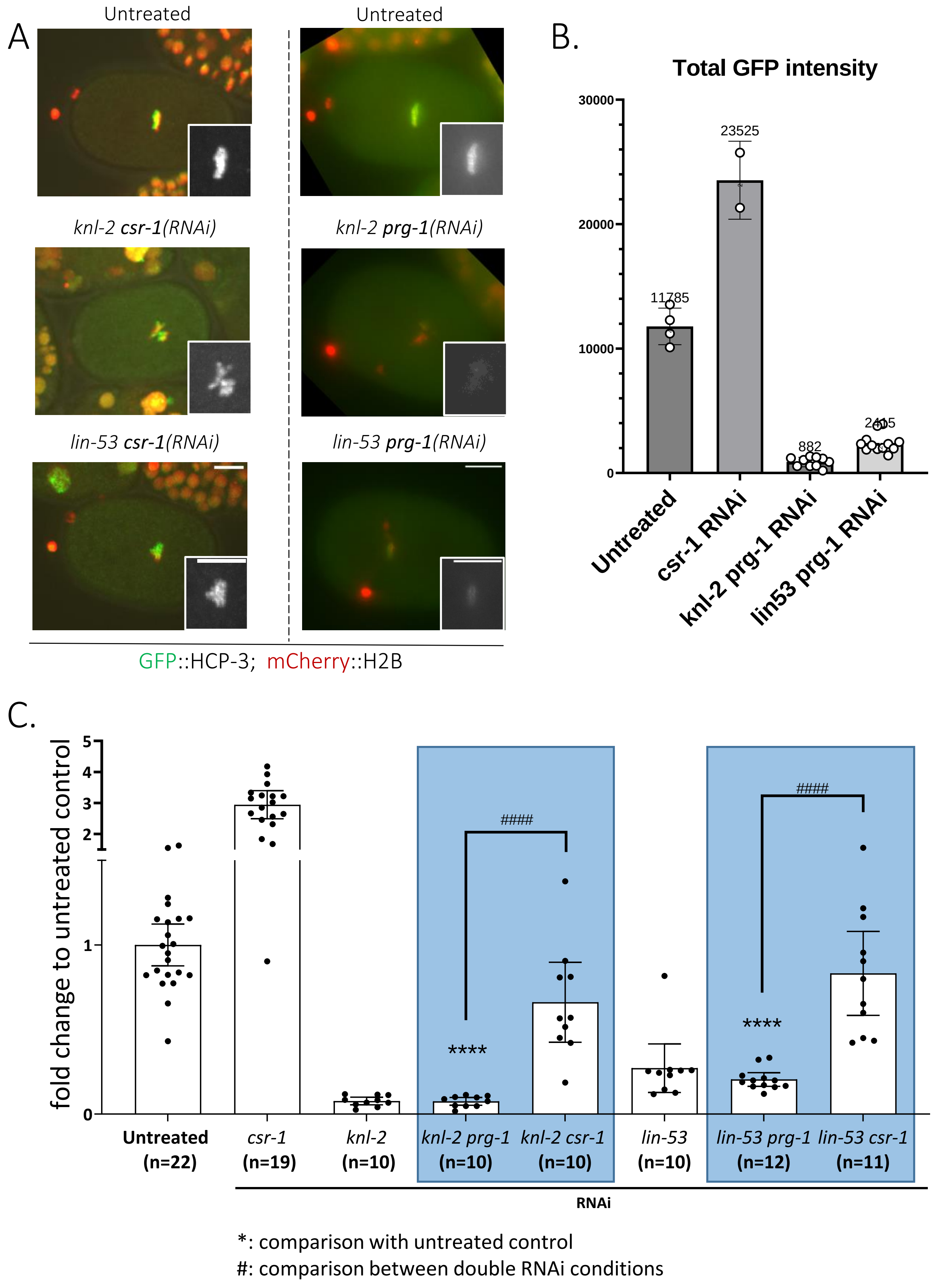

Journal of Cell Science • Supplementary information

Fig. S6. Double knockdown control *knl-2 prg-1*(RNAi) or *lin- 53 prg-1*(RNAi) do not trigger HCP-3 increase on chromatin.

(A) One-cell embryos at metaphase with different RNAi treatment. Double RNAi were performed by mixing the dsRNA in 1:1 ratio and injected to L4 larvae. The image on the left of untreated, *knl-2 csr-1 (RNAi)*, *lin-53 csr-1 (RNAi)* are extracted from Figure 5. Image on the right showing the double knockdown control knocking down another Argonaute *prg-1* instead of *csr-1*. Scale bars: 10 μm in 1-cell embryo image and 5 or 10 μm in the enlarged chromatin insets. After quantified the total GFP intensities (B), each data points were divided by the mean untreated control intensity to obtain the fold change to control to compare with other treatments (C). ****/####: $p<0.0001$, unpaired t-test. n indicates number of one-cell metaphase used for quantification. Error bars: Stand deviation in (B), 95% confidence interval in (C).

Journal of Cell Science • Supplementary information

Table S1. Strains used in this study

Strain	Genotype	Source	Figure
N2	wild type (ancestral N2 Bristol)	CGC	2B, 4B, S1C, S1F, S4A
AV221	*unc-119(ed3) meT8 (III); meIs4 [lacOp/rol-6(su1006)::lacO] meT8 (IV); mels1 [pie-1p/GFP::lacI; unc-119(+)]*	CGC	S1D
OD421	*unc-119(ed3) III; ltSi4 [pOD833; hcp-3p/GFP::hcp-3; cb unc-119(+)] II; hcp-3(ok1892) III; ltIs37[pAA64; pie-1p/mCherry::his-58; unc-119 (+)]*	Oegema & Desai lab	2A, 3-6, S1A-B, S2-3, S4A, S5-6
TH32	*unc-119(ed3)III; ruIs32 [pie-1p::GFP::H2B + unc-119(+)] III. ddIs6 [tbg-1::GFP + unc-119(+)] V.*	CGC	1
OD87	*ltIs4[pIC32; Ppie-1::GFP-TEV-STag::mis-12; unc-119 (+)]; unc-119(ed3) III?; ltIs37[pAA64; Ppie-1::mCherry::his-58; unc-119 (+)] IV*	Oegema & Desai lab	2A
OD405	*unc-119(ed3) III; ltSi1[pOD809/pJE110; Pknl-1::KNL-1reencoded::RFP; cb-unc-119(+)] II, ltIs3[pIC31; Ppie-1 ::hcp-1::GFP-TEV-STag + unc-119(+)]*	Oegema & Desai lab	2A
OD86	*unc-119(ed3) III; ltIs1 [pIC22; pie-1/GFP::knl-3; unc-119 (+)]; ltIs37 [pAA64; Ppie-1::mCherry::his-58; unc-119 (+)] IV*	Oegema & Desai lab	2A
OD1174	*ltSi386[pOD1344/pAG36; Phcp-3::GFP::hcp-3(reencoded); cb-unc-119(+)]I; unc119(ed3)III?*	Oegema & Desai lab	-
VC1393	*hcp-3(ok1892) III/hT2 [bli-4(e937) let-?(q782) qIs48] (I;III)*	CGC	-
WYY31	*ltSi386[pOD1344/pAG36; Phcp-3::GFP::hcp-3(reencoded); cb-unc-119(+)]I; unc119(ed3)III; hcp-3(ok1892) III/hT2[bli-4(e937) let-?(q782) qIs48](I;III)*	Generated in this study by crossing OD1174 with VC1393	S4B
OD1231	*ltSi386[pOD1344/pAG36; Phcp-3::GFP::hcp-3(reencoded); cb-unc-119(+)]I; ltSi242[pOD1267/pAG31; Pcsr-1::csr-1(reencoded; D606A, D681A: isoform b numbering), cb-unc-119(+)]II; unc-119(ed3)III?*	Oegema & Desai lab	6E
OD1232	*ltSi386[pOD1344/pAG36; Phcp-3::GFP::hcp-3(reencoded); cb-unc-119(+)]I; ltSi240[pOD1265/pAG29; Pcsr-1::csr-1(reencoded); cb-*	Oegema & Desai lab	6E
	unc-119(+)]II; unc-119(ed3)III?		

Journal of Cell Science • Supplementary information

Table S2. Oligo used in this study

For dsRNA production:

Targeted gene	Forward/ Reverse	Primer sequence (5' to 3')	Product size (bp)
csr-1 (F20D12.1)	Forward	TAATACGACTCACTATAGGGgagaagcgtgactggaaagcac	944
	Reverse	TAATACGACTCACTATAGGGagcggctgtcttaatggtttgc	
zyg-1	Forward	AATTAACCCTCACTAAAGGtggacggaaattcaaacgat	1055
	Reverse	TAATACGACTCACTATAGGaacgaaattcccttgagctg	
knl-1	Forward	TAATACGACTCACTATAGGGgaagccgctgacaaatcaacga	1261
	Reverse	TAATACGACTCACTATAGGGcaacgttgaccacagctggatc	
knl-2	Forward	AATTAACCCTCACTAAAGGgctcaaaaccggctaattca	516
	Reverse	TAATACGACTCACTATAGGtgatcagcagccacatcttc	
lin-53	Forward	AATTAACCCTCACTAAAGGccccgttcttgtacgatctc	971
	Reverse	AATTAACCCTCACTAAAGGgcagattggtcttctccaa	
hcp-3	Forward	AATTAACCCTCACTAAAGGcaaaatgagagcgtcacaa	829
	Reverse	TAATACGACTCACTATAGGtttcgaccaaaaatgcttcc	
ego-1	Forward	AATTAACCCTCACTAAAGGatctgtcgcccactcatttc	804
	Reverse	AATTAACCCTCACTAAAGGtctcgaatcgtcccaaattc	
csr-1 isoform a	Forward	TAATACGACTCACTATAGGGcggataatgtcggtcgtggc	368
	Reverse	TAATACGACTCACTATAGGGtctcccgcgatagtttccgc	
hrde-1	Forward	TAATACGACTCACTATAGGGgcttcgaattgctccaggcc	558
	Reverse	TAATACGACTCACTATAGGGtccggcttcttctcccttgc	
csr-1 (endogenous-specific, Figure 6)	Forward	TAATACGACTCACTATAGGGgtagcaggttatactcgaact	419
	Reverse	TAATACGACTCACTATAGGGccgcatttaatgttggcttt	
klp-7	Forward	AATTAACCCTCACTAAAGGacgagctggtctatcgcttcac	662
	Reverse	AATTAACCCTCACTAAAGGgctgtctgaacgggacatgtgag	

Journal of Cell Science • Supplementary information

For RT-PCR:

**Targeted gene**	**Forward/Reverse**	**Primer sequence (5' to 3')**	**Product size (bp)**
act-2	Forward	CTTGCCCCATCAACCATGAAGATCA	155
	Reverse	TGAACAATTGATGGTCCAGACTCG	
hcp-3	Forward	AGCGTCACAAGGATCATGCA	164
	Reverse	TGCGCTTCAGTGTAGTCGTT	
csr-1	Forward	CCAGTGCAAGGTACTGCAAAGG	188
	Reverse	TAAAGCCCGGTTCCTCTCTTGG	
csr-1 *isoform a*	Forward	TCAGAGGTGGACGAGGAGGT	157
	Reverse	ACGGTAGTGGTTTGGGTCGA	
hrde-1	Forward	GACAAGGGAGAACGCGAGGT	168
	Reverse	GTTGACCTTGATCCGCTGGC	

Table S3. Antibodies used in this study

**Antibody (against)**	**Host**	**Dilution**	**Source**	**Used in Figure**
GFP	Rabbit	1:1000	Novus (NB600-308)	4B, S1C, S4A
α-Tubulin	Mouse	1:3000	Abcam (ab7291)	4B, S1C, S4A
LacI	Mouse	1:500	Millipore (05-503)	S1D
AIR-2	Rabbit	1:1000	Gift from OD lab	S1E
HCP-3	Rabbit	1:1000	Novus (29540002)	2B
KNL-2	Rabbit	1:1000	Gift from OD lab	2B
NDC-80	Rabbit	1:1000	Gift from OD lab	2B
HCP-4	Rabbit	1:1000	Gift from OD lab	2B
MIS-12	Rabbit	1:1000	Novus (35550002)	2B
Histone H4	Rabbit	1:500	Abcam (ab10158)	3
GFP	Mouse	1:500	Abcam (ab1218)	3

Journal of Cell Science • Supplementary information

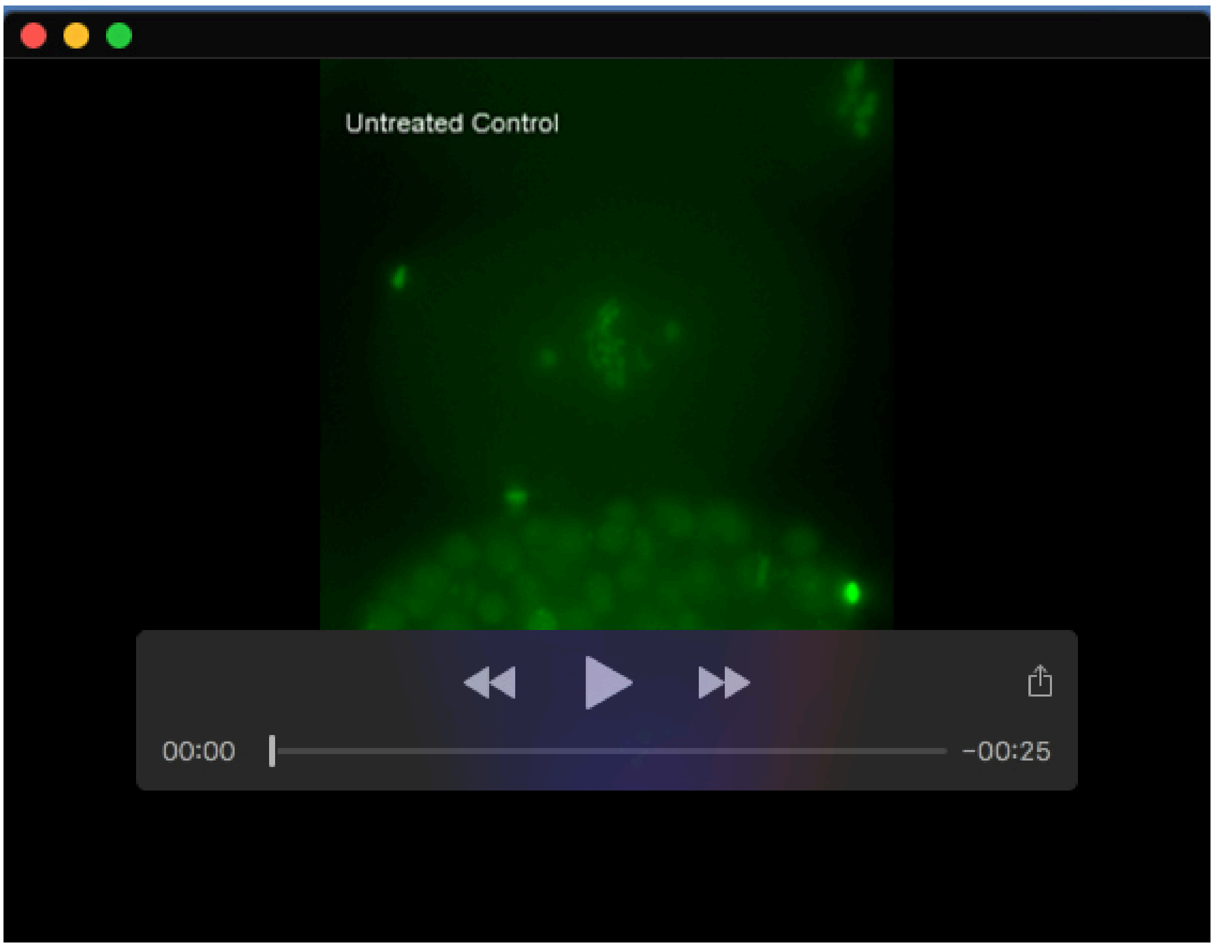

Movie 1. First cell division in untreated TH32. GFP::H2B; GFP::Gamma-tubulin, 30-sec time interval, 2fps.

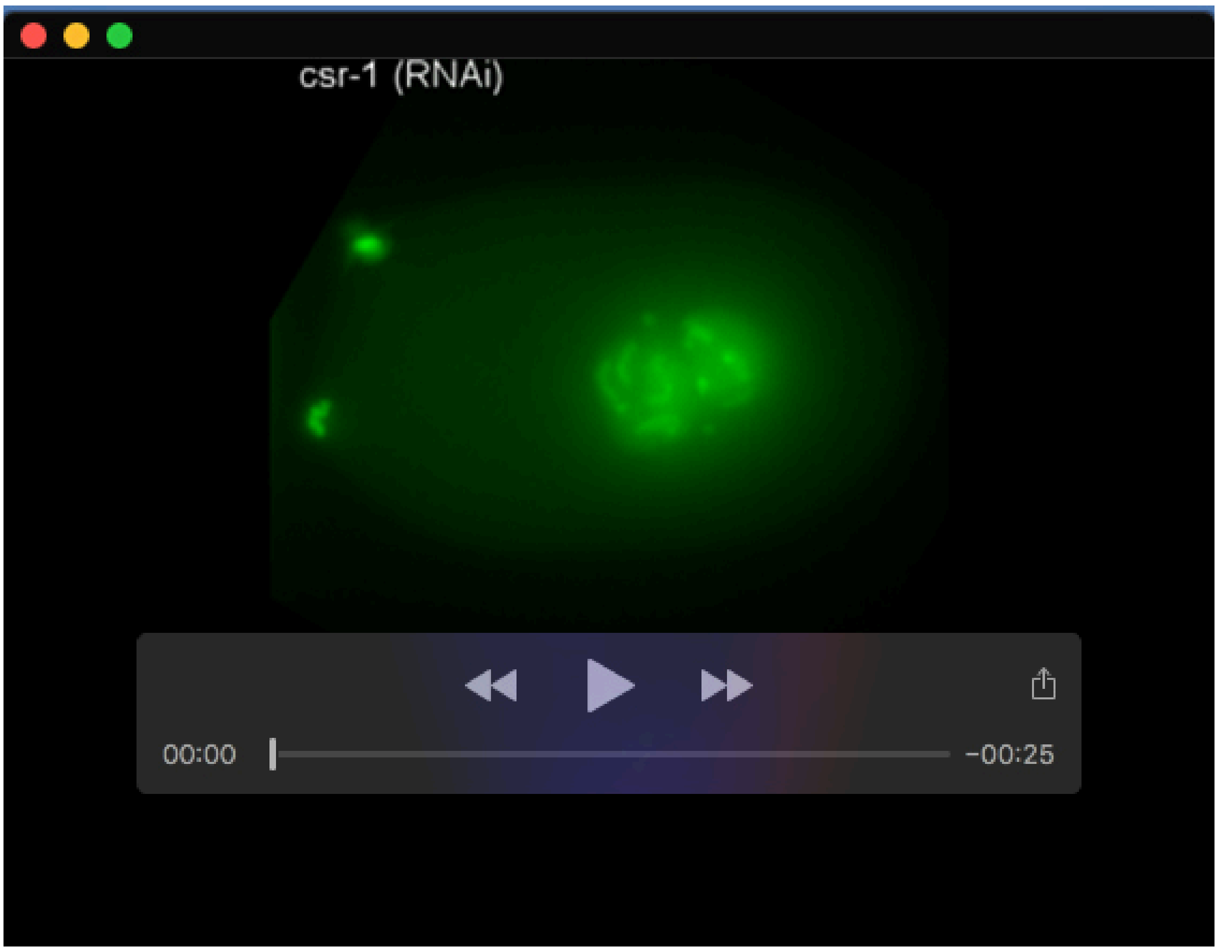

Movie 2. First cell division in *csr-1*(RNAi) TH32 showing bridging chromatin at anaphase. GFP::H2B; GFP::Gamma-tubulin, 30-sec time interval, 2fps.

Journal of Cell Science • Supplementary information
