# Supplementary material for: Argonaute protein CSR-1 restricts localization of holocentromere protein HCP-3, the C. elegans CENP-A homolog
Source: J Cell Sci. 2024 Sep 18;137(18):jcs261895. doi: 10.1242/jcs.261895 (PMC11423810; doi:10.1242/jcs.261895)
Supplement: Supplementary file 1 [file joces-137-261895_review_history.pdf]

Argonaute protein CSR-1 restricts localization of holocentromere protein HCP-3, the *C. elegans* CENP-A homolog

Charmaine Yan Yu Wong, Hok Ning Tsui, Yue Wang and Karen Wing Yee Yuen
DOI: 10.1242/jcs.261895

Editor: Renata Basto

Review timeline
Original submission: 17 December 2023
Editorial decision: 23 January 2024
First revision received: 23 May 2024
Accepted: 11 July 2024

Original submission

First decision letter

MS ID#: JOCES/2023/261895

MS TITLE: CSR-1 RNA interference pathway restricts holocentromere protein CENP-A/HCP-3 localization

AUTHORS: Charmaine Yan Yu Wong, Hok Ning Tsui, Yue Wang, and Karen Wing Yee Yuen
ARTICLE TYPE: Research Article

We have now reached a decision on the above manuscript.

To see the reviewers' reports and a copy of this decision letter, please go to: https://submit-jcs.biologists.org and click on the 'Manuscripts with Decisions' queue in the Author Area. (Corresponding author only has access to reviews.)

As you will see, the reviewers raise a number of substantial criticisms that prevent me from accepting the paper at this stage. They suggest, however, that a revised version might prove acceptable, if you can address their concerns. If you think that you can deal satisfactorily with the criticisms on revision, I would be pleased to see a revised manuscript. We would then return it to the reviewers.

Please ensure that you clearly highlight all changes made in the revised manuscript. Please avoid using 'Tracked changes' in Word files as these are lost in PDF conversion.

I should be grateful if you would also provide a point-by-point response detailing how you have dealt with the points raised by the reviewers in the 'Response to Reviewers' box. Please attend to all of the reviewers' comments. If you do not agree with any of their criticisms or suggestions please explain clearly why this is so.

Reviewer 1

Advance summary and potential significance to field

In this research article, Yan Yu Wong et al. analyse the role of the Argonaute protein CSR-1 in the localization of CENP-A/HCP-3 at holocentromeres in the C. elegans zygote. Employing a

© 2024. Published by The Company of Biologists under the terms of the Creative Commons Attribution License (https://creativecommons.org/licenses/by/4.0/).

combination of genetic perturbations with microscopic observations, the authors first confirmed previously published findings on the role of CSR-1 in proper chromosome alignment and segregation in C. elegans zygotes. However, in contrast to previous publications, they attribute these defects to the ectopic over-recruitment of CENP-A/HCP-3, and downstream outer kinetochore components, at holocentromeres upon CSR-1 loss of function. By analyzing CENP-A/HCP-3 distribution on stretched chromatin fibers, the authors show that in absence of CSR-1, CENP-A/HCP-3 is primarily mislocalized at ectopic centromeric foci. The authors then show that this mislocalization is not associated to an effect of CSR-1 depletion on HCP-3 mRNA or protein levels, nor to a change in HCP-3 turnover at holocentromeres. Furthermore, HCP-3 over-recruitment could only partially be functionally linked to the CENP-A/HCP-3 loading factors LIN-53 and KNL-2. This last result implies the existence of a 'non-canonical' mechanism of CENP-A/HCP-3 loading at holocentromeres in C. elegans zygotes. Finally, Yan Yu Wong et al. show that the CENP-A/HCP-3 over-recruitment phenotype could be specifically attributed to the germline-expressed 'b' isoform of csr-1 (and not to the somatic 'a' isoform) and specifically to its 'slicing' activity, and that it could be recapitulated by depleting the RdRp EGO-1 (although the reported effect is extremely mild) suggesting that the associated RNAi pathway could be involved in the observed phenotype.
In conclusion, this study offers intriguing, but interesting, new findings that both align with and, to some extent, diverge from earlier publications regarding the role of CSR-1 in regulating accurate chromosome segregation.

Comments for the author

Here are specific points to address before publication:
Major points:
1/ Gerson-Gurwitz et al. (2016) analyzed the effect of CSR-1 depletion on HCP-3 intensity at holocentromeres in C. elegans zygotes (Figure 2D) and reported no discernible difference compared to control zygotes. This observation contradicts the primary outcome of the current study. A revised version of the manuscript should at least thoroughly discuss potential explanations for this discrepancy.

2/ The images depicted in Figure 2B do not match the corresponding quantifications. For instance, the visual assessment of KNL-2 intensity in the images of untreated and csr-1(RNAi) zygotes does not appear markedly different, contrary to what the bottom graph implies. Additionally, HCP-4 seems dimmer in the csr-1(RNAi) image, in contrast to the quantification that suggests the opposite trend. This issue could either be caused by a poor choice of representative images or to an issue with image scaling, and should be fixed.

3/ In Figure 3A, the DAPI and H4 signals appear noticeably more intense in the illustrated csr-1(RNAi) fiber compared to controls. Furthermore, a discernible local correlation between both signals and GFP::HCP-3 is evident. Can the authors confirm with certainty that single chromatin fibers are consistently analyzed in both conditions? It is crucial to quantitatively establish the absence of correlation between the DAPI/H4 signals and the GFP::HCP-3 signal in order to ensure the reliability of the results.

4/ Figure 6 is not organized or cited as described in the text. This should be fixed. Furthermore, the impact of EGO-1 depletion appears to be relatively mild, even with a substantial number of analyzed embryos (78 embryos, compared to the typical 4 to 8 embryos in other conditions). I would thus tone down conclusions drawn from these results.

5/ The sample size is notably small in several instances, particularly evident in Figure 5 and 6 where fewer than 9 embryos, and sometimes as little as 3 embryos, were analyzed per condition. Typically, comparable studies analyze a larger number of embryos (at least 10 embryos per condition) to prevent overemphasizing the significance of potential outliers.

Minor points:
1/ A general spelling check and rewriting certain sections would significantly improve the overall quality of this manuscript.

2/ Figure 7 and 8 could be improved (for example, in Figure 7, green is not the same in untreated and csr-1(RNAi), the thickness of chromosomes is also different, etc) and should be merged.

© 2024. Published by The Company of Biologists under the terms of the Creative Commons Attribution License (https://creativecommons.org/licenses/by/4.0/).

3/ In Figure 8, 'Factors that we have tested to be not required or negative are shown in red.' does not refer to anything visible on the figure. Moreover, unless I overlooked something, doesn't the fact that the SIN mutant of CSR-1 recapitulates the full loss-of-function phenotype exclude hypothesis 2 ('bind target DNA loci')?

Reviewer 2

Advance summary and potential significance to field

CSR-1 is an Argonaute protein that binds 22G RNAs, an abundant class of small RNAs primarily found in the C. elegans germline. The first phenotypic analysis of csr-1 mutants in 2006 included the observation of chromosome segregation defects, suggesting a connection between the small RNA machinery and the centromere. Since then, several studies have investigated whether CSR-1 directly influences centromeres, or whether loss of CSR-1 triggers a cascade of misregulation that eventually also affects the centromere, but none of these studies conclusively pinpointed the mechanistic link between CSR-1 and centromeres.

The current manuscript by Wong et al revisits the analysis of the centromere-specific histone variant HCP-3 in the context of csr-1 depletion. The experiments are of high quality and well documented. However, most of the results in this manuscript recapitulate findings that have already been reported in previous publications that are all cited. The chromosome segregation defects have been known since 2006 (Yigit et al., Cell) and have been thoroughly described by Claycomb et al., Cell, 2009. Another study (Gerson-Gurwitz, Cell, 2016) quantified centromere biorientation in absence of csr-1 and the effect on kinetochore proteins, and explained how CSR-1 links to kinetochore function. Increased levels of HCP-3 on chromatin in absence of CSR-1 was shown by Ladouceur et al., JCB, 2017. The similar phenotypes of csr-1 of ego-1 knockdown were described by Claycomb et al., 2009. The comparison of the effects of knockdown of isoform a vs knockdown of isoforms a and b, and effects of the CSR-1 SIN mutant on chromosome segregation were presented by Gerson-Gurwitz et al., 2016.

The authors present some new approaches, but the results in this current study still mainly recapitulate and confirm previous findings, and they fall short of providing new mechanistic insight, and do not clarify the role of CSR-1 in the regulation of holocentromeres. There are aspects of the manuscript that are new, including the analysis of HCP-3 foci on stretched chromatin fibers, or the analysis of the co-depletion of csr-1 with knl-2, lin-53, prg-1 or hrde-1. However, these results are premature and in the current form do not significantly advance our understanding of the link between CSR-1 and holocentromere regulation. They are not sufficiently controlled to draw strong conclusion (see major comments below).

Comments for the author

Major concerns

The manuscript overstates the findings and their significance. For example, there is no data about the RNA interference pathway, as stated in the title, and the link to small RNAs is only implied by the fact that CSR-1 binds small RNAs. In the discussion, the authors claim that “Our study has shed light on how holocentromeric regions are regulated in C. elegans, and exemplified how a RNAi pathway can be involved in the determination of centromere regions”, but the authors neither analyze holocentromeric regions, nor RNAi pathways.

Co-depletion of mRNAs of multiple genes by RNAi is not always comparable with single RNAi treatment. The authors should show the efficiency of the knockdown systematically, as for csr-1 and hrde-1 depletion in Figure S4B. In the hrde-1 example, the RNAi treatment was effective, but clearly less so in the co-depletion experiment than in the single depletion experiments. Without these controls, it is not possible to draw the conclusion from the experiments in Figure 5 that “CSR-1 promotes HCP-3 chromatin localization in embryos depleted of HCP-3 loading factors”.

© 2024. Published by The Company of Biologists under the terms of the Creative Commons Attribution License (https://creativecommons.org/licenses/by/4.0/).

In the stretched chromatin fiber experiment, all fibers, and not just one example, should be shown. Since the fibers likely represent different genomic regions, a direct comparison between wildtype control and csr-1 depletion is difficult. The increased number of foci is clearly visible, but it is impossible to conclude if this reflects an increase of HCP-3 at centromeric regions, or indeed a spread to additional genomic regions, as the authors propose.

Reviewer 3

Advance summary and potential significance to field

In this manuscript, Wong and coauthors provide evidence that the argonaute ortholog CSR-1 plays a critical role in the localization of the kinetochore determinant HCP-3 to chromosomes. While there are many potential ways this control could work in principle, the authors show that it is not through altering transcriptional or translational dynamics of HCP-3, nor its turnover on chromosomes. While the precise mechanism still cannot be identified, the authors demonstrate that the slicer activity of CSR-1 isoform b may indirectly restrict the access of HCP-3 to chromatin either by somehow altering its loading factors or by epigenetic modification to chromatin. Since the well-characterized loading factors KNL-1 and LIN-53 do not seem to change in *csr-1* knockdowns, however, the former possibility would involve CSR-1 operating through an as-yet unidentified pathway. Several lines of evidence are shown for their conclusions; in particular the use of extended fiber imaging provides a plausible and convincing explanation for the appearance of higher HCP-3 intensity on chromosomes without concomitant increase in expression. Overall, this manuscript advances our understanding by clarifying the potential control mechanisms of HCP-3 recruitment.

Comments for the author

The manuscript's data are of high quality and are adequately controlled. Some comments on particular points follow that ought to be resolved before publication:

- Several figures such as 2A show different images with different intensities, and conclusions are drawn based on the intensity being greater in one condition. For all such experiments, it should be stated explicitly whether the microscopy conditions were the same (for example, excitation light intensity and capture time, or antibody concentrations if appropriate) as they ought to be to interpret the results.

- Some conclusions are drawn from double-RNAi experiments (Fig.1 C knl-1 csr-1(RNAi); however, since targeting two genes by RNAi often leads to sub-optimal knockdown, a test of RNAi effectiveness for lin-53 csr-1 and knl-2 csr-1 should be performed as it was for csr-1 hrde-1 (Figure S4).

- Fig. 2B : Please add detail in methods section explaining how line profiles were drawn and analyzed. In particular, how were the areas for line profiles selected (and how was bias in selection avoided)?

Additional minor comments:
- Fig.1A, 3A, 4C - Please consider providing grayscale images instead of single-channel color images, for better contrast. Especially, the blue DAPI channel is very difficult to see.

- 4C: Bar graphs at bottom can be made larger so labels are bigger/more legible.

- Line 491: "(Spot Analysis)" - Please provide more detail about what kind of software was used for this analysis and how it was used.

- The legends and figures for Supplemental Figures S2 and S3 are out of order.

- Figure 8: the legend states "Factors that we have tested to be not required or negative are shown in red." but no such factors seem to be listed.

© 2024. Published by The Company of Biologists under the terms of the Creative Commons Attribution License (https://creativecommons.org/licenses/by/4.0/).

First revision

Author response to reviewers' comments

MS ID#: JOCES/2023/261895

[Reviewer 1] Comments for the Author:

Here are specific points to address before publication:

Major points:

1. **Gerson-Gurwitz et al. (2016) analyzed the effect of CSR-1 depletion on HCP-3 intensity at holocentromeres in C. elegans zygotes (Figure 2D) and reported no discernible difference compared to control zygotes. This observation contradicts the primary outcome of the current study. A revised version of the manuscript should at least thoroughly discuss potential explanations for this discrepancy.**

➢ Thanks for Reviewer 1 to point out this. We apologize for not addressing this clearly in the last version of the manuscript. Our results were in fact not contrasting with those in Gerson-Gurwitz et al, Cell, 2016, but were analyzed differently to emphasize a different conclusion. In their immunofluorescence experiment (Fig. 2D) they normalized the HCP-3 signal intensity in each prometaphase chromosome linescan, such that the peak intensity is set to one, in order to look at the pattern of the holocentromeres across the transverse plane of the condensed chromosome. On the other hand, we compared the intensity change without internal normalization in our linescans between the *wildtype* and the *csr-1*(RNAi) (imaged under exactly same conditions) to emphasize the absolute increase in HCP-3 level on the chromatin. In our hands, the distribution pattern of HCP-3 in *csr-1*(RNAi) has not changed, consistent with Gerson-Gurwitz et al, Cell, 2016. We have personal communications with Gerson-Gurwitz and the Desai lab to verify this observation of higher HCP-3 intensity in *csr-1*(RNAi) during the preparation of our work. They explained that they also observed the increase of HCP-3 on chromatin, but their study did not focus on the change in HCP-3 level, and thus chose to display the linescan quantification using a relative scale.

Now we have made it more clear by adding a discussion introducing Gerson-Gurwitz 2016's work on this, compare the differences in aim, emphasis, and analysis approach, but also highlighting the similarity in the results (line 273), "Overall, the level of HCP-3 and kinetochore proteins signal on prometaphase chromosomes significantly increased in csr-1(RNAi) embryos, while the centromere and kinetochore proteins remain concentrated on the poleward faces on the chromosomes. This is similar to the linescan results reported by Gerson-Gurwitz et al. In their study, each linescan of HCP- 3 signal was normalized to its own peak intensity to study the pattern and distribution of holocentromeres across the transverse plane of the condensed chromosome. On the other hand, our analysis focused on the absolute intensity change in csr-1(RNAi) embryos rather than the normalized distribution pattern."

2. **The images depicted in Figure 2B do not match the corresponding quantifications. For instance, the visual assessment of KNL-2 intensity in the images of untreated and csr-1(RNAi) zygotes does not appear markedly different, contrary to what the bottom graph implies. Additionally, HCP-4 seems dimmer in the csr-1(RNAi) image, in contrast to the quantification that suggests the opposite trend. This issue could either be caused by a poor choice of representative images or to an issue with image scaling, and should be fixed.**

➢ Regarding the discrepancy between the images depicted in Figure 2B and the corresponding quantification, we apologize for any confusion caused by the display of representative images. We would like to point out that for KNL-2, the quantification of the image shows similar levels of protein localization on the chromosomes in both the

© 2024. Published by The Company of Biologists under the terms of the Creative Commons Attribution License (https://creativecommons.org/licenses/by/4.0/).

untreated and *csr-1*(RNAi). Thus, the image aligns with our conclusion.

We have made the following changes to address this:

1. We have selected representative images in Figure 2B that reflect the average intensity. Indeed, the image scaling for HCP-4 was not equal and is now fixed.
2. We have added an example image to illustrate the specific lines we drew on the chromosome for linescan analysis. We hope this provides a clearer visual representation of the analyzed region.
3. In the results (line 160), we have stated more clearly that KNL-2 intensity, thus its chromosomal localization, is similar in the untreated and csr-1(RNAi). "Among the proteins quantified (Figure 2B), the levels of centromeric protein HCP-3 and the kinetochore proteins HCP-4 and NDC-80, but not the centromere licensing factor Mis18BP1 homolog KNL-2, increased at the poleward-faces of the chromosomes in csr-1(RNAi) embryos."
4. In the materials and methods (line 483), we have stated the criteria used to select the chromosomes for analysis, including factors such as ensuring the chromosome lie flat and have minimal rotation along the imaged x-y plane. "Our inclusion criteria of an analysable chromosome is: choosing cells with condensed chromosomes and not (prometaphase); choosing chromosomes that lie flat (the two centromeres on the xy plane instead of rotated e.g. x-z plane or y-z plane). Please refer to supplementary document for an example and illustration of the chromosomes qualified."
5. In the figure legend of Figure 2B, we have included a statement indicating that the comparison is made using chromosomes from the same cell-stage for a certain protein, emphasizing the consistency in the experiment. "Fixed embryos at the same cell stage were used to compare the protein distribution and level on condensed chromosomes between untreated control, and the csr- 1(RNAi) (HCP-3: 2-cell stage, KNL-2: 2-cell stage, HCP-4: 1-cell stage, NDC-80: 2-cell stage)."

3. **In Figure 3A, the DAPI and H4 signals appear noticeably more intense in the illustrated csr-1(RNAi) fiber compared to controls. Furthermore, a discernible local correlation between both signals and GFP::HCP-3 is evident. Can the authors confirm with certainty that single chromatin fibers are consistently analyzed in both conditions? It is crucial to quantitatively establish the absence of correlation between the DAPI/H4 signals and the GFP::HCP-3 signal in order to ensure the reliability of the results.**

➢ We appreciate the reviewer's comments and insightful observations regarding our stretched chromatin fiber experiment. We acknowledge the need to include images of multiple fibers for readers to comprehend. In our revised manuscript, we have included additional example images of the stretched chromatin fibers from both the *wildtype* control and *csr-1* depletion conditions in Figure 3A and Figure S6. We hope this will enable a better understanding of the overall trends and variations within our experimental data.

The chromatin were stretched to a slightly different degree along the fiber and across samples even if cautions have been taken to pull the slides at a relatively reproducible speed. To address such variation in our quantification, we normalized the foci intensity and density (nearest neighbor distance) to the H4 intensity measured in the same ROI. For foci intensity, we quantify the relative intensity of GFP::HCP-3 (i.e. GFP/H4) of each ROI (Figure 3D). For foci density, apart from the unnormalized NND we show in Figure 3F, we developed a method to normalize NND for each ROI (i.e. NND/ inverse of H4 intensity) and show as supplementary in Figure S2E. The neighboring distance of a given ROI is expected to reduce if the fiber is thicker, or in other words, stretched less (Figure S2D). After taken the H4 intensity of the foci into account, the normalized NND in *csr-1*(RNAi) is consistently smaller than the untreated control. This normalization method is stated in our Materials and methods under "Chromatin fiber preparation and analysis of the foci ROI".

Despite the general trend where more stretching leads to both lower H4 and lower GFP::HCP-3, we have showed by a scatter plot of the intensities that for a given H4 intensity, the GFP::HCP-3 intensity is higher in csr-1 knockdown condition (Figure S2C). Similarly, for a given H4 intensity, the NND of the foci is smaller in csr-1 knockdown condition (Figure S2D).

4. **Figure 6 is not organized or cited as described in the text. This should be fixed. Furthermore, the impact of EGO-1 depletion appears to be relatively mild, even with a**

© 2024. Published by The Company of Biologists under the terms of the Creative Commons Attribution License (https://creativecommons.org/licenses/by/4.0/).

substantial number of analyzed embryos (78 embryos, compared to the typical 4 to 8 embryos in other conditions). I would thus tone down conclusions drawn from these results.

➢ We have corrected the figure label for Figure 6: (A) EGO-1; (B) CSR-1 isoforms; (C) PRG-1 piRNA (D) HRDE-1 (E) CSR-1 SIN mutant.

Regarding the impact of EGO-1 depletion (Figure 6A), we agree that how the ego-1(RNAi) carried out were different from the other sets and wasn't properly compared. In our previous experiment, the EGO-1 knockdown was performed by dsRNA soaking). We have conducted a new experiment to replace the previous one.

In this new experiment, we knocked down *ego-1* by dsRNA injection and quantified metaphase intensity in one-cell stage embryos, which is the same condition used in other panels of Figure 6. Notably, this revised experiment consistently resulted in an increase in GFP::HCP-3 levels by over 2 folds when compared to the untreated control. We have replaced this in the revised manuscript to ensure the accuracy and clarity of our conclusions regarding the role of EGO-1 in regulating GFP::HCP-3 expression.

5. The sample size is notably small in several instances, particularly evident in Figure 5 and 6 where fewer than 9 embryos, and sometimes as little as 3 embryos, were analyzed per condition. Typically, comparable studies analyze a larger number of embryos (at least 10 embryos per condition) to prevent overemphasizing the significance of potential outliers.

➢ Regarding the small sample size in certain instances, particularly in Figure 5 and 6, we agree with the reviewer's point that it is important of analyzing a larger number of embryos to ensure the robustness of the results and to minimize the impact of potential outliers.

We have increased the sample size to be equal to or greater than 10 embryos per condition for the panels mentioned in Figures 5 and 6 (for the GFP::HCP-3 metaphase intensity under different perturbations), and Figure 1B (for AB, P1 cells mitotic timing), Figure 2A (for the metaphase intensity of HCP-3 in untreated and *csr-1*(RNAi); MIS-12 in *csr-1*(RNAi). We have observed smaller standard deviations in most of the assays, while the conclusions of the study remain consistent. For tracking spindle pole separation which we presented as Figure 1C previously, we have increased the sample size, but we no longer observe a significant delay in *csr-1*(RNAi). So we now reported this and moved this part to Supplementary Figure S1E. Nonetheless, this doesn't affect our main conclusion that CSR-1 is important for regulating chromatin HCP-3 level.

Additionally, we have made adjustments to the presentation of the data by changing the y-axis from raw GFP intensity (a.u.) to "fold change of the fluorescence intensity to untreated control average" for Figure 2A, 5, 6. This normalization method provides a relative measure of the fluorescence intensity, making it easier to compare across different samples and samples analyzed at different times.

With the increased sample size and the normalization method, we believe that these improvements address the concern and enhance the reliability of the results.

Minor points:

1. A general spelling check and rewriting certain sections would significantly improve the overall quality of this manuscript.

➢ Thank you for your feedback. We conducted a thorough spell check and rewrote sections of the manuscript, including the introduction, results, discussion, and materials and methods, to improve the overall quality.

2. Figure 7 and 8 could be improved (for example, in Figure 7, green is not the same in untreated and csr-1(RNAi), the thickness of chromosomes is also different, etc) and should be merged.

➢ The difference in color depth and thickness were meant to imply the difference in HCP-3 level on chromosome. We have changed the way we represent the HCP-3 density on the poleward face of chromosomes in Figure 7. We now use denser green circles instead of a darker green color and thickness to illustrate the differences in untreated and *csr-1*(RNAi).

© 2024. Published by The Company of Biologists under the terms of the Creative Commons Attribution License (https://creativecommons.org/licenses/by/4.0/).

We now merge figure 7 and 8 as one figure to summarize the knockdown effect of CSR-1 and the requirement for CSR-1 to repress HCP-3.

3. In Figure 8, 'Factors that we have tested to be not required or negative are shown in red.' does not refer to anything visible on the figure. Moreover, unless I overlooked something, doesn't the fact that the SIN mutant of CSR-1 recapitulates the full loss-of-function phenotype exclude hypothesis 2 ('bind target DNA loci')?

We have removed the statement from the figure legend to match the figure.

Regarding the second point raised, we agree that the SIN mutant of CSR-1 exhibits a similar effect on HCP-3 localization as the full knockdown phenotype. However, it is important to note that we still do not understand how the SIN mutation affects CSR-1 function. In a recent study, Singh et al., performed IP-RNAseq to show that the catalytically inactive CSR-1 mutant-bound siRNA profile changed [1]. Also, CSR-1's slicer activity is shown to be required for its chromatin-related functions, as mentioned in the study by Wedeles et al. (2013). These results suggest that in CSR-1 SIN mutant, the CSR-1 targets, including its targets on DNA loci, may be altered.

Therefore, we think this result is not sufficient to exclude our hypothesis 2 that CSR-1 affects HCP-3 via binding target DNA loci.

In discussion (line 336), we added "The PIWI domain of CSR-1, which controls its target cleavage and binding functions, also appears to play a role in restricting HCP-3 localization. Mutation at the PIWI domain abolishes CSR-1's ability to cleave the target (Aoki et al., 2007), and globally affects the abundance and binding profile of CSR-1-associated 22G-siRNAs (Singh et al., 2021). Besides impacting transcript regulation, the PIWI domain also interacts with protein cofactors, such as glycine-tryptophan (GW) repeat proteins that can repress translation (Liu et al., 2005, Eulalio et al., 2008). Therefore, it is possible that CSR-1 affects HCP-3 localization through PIWI domain-mediated interactions with regulatory factors."

*****

[Reviewer 2] Comments for the Author:

Major concerns

The manuscript overstates the findings and their significance. For example, there is no data about the RNA interference pathway, as stated in the title, and the link to small RNAs is only implied by the fact that CSR-1 binds small RNAs. In the discussion, the authors claim that "Our study has shed light on how holocentromeric regions are regulated in C. elegans, and exemplified how a RNAi pathway can be involved in the determination of centromere regions", but the authors neither analyze holocentromeric regions, nor RNAi pathways.

➢ We appreciate the reviewer's concerns regarding the potential overstatement of our findings and the generalization of the CSR-1 effect to its RNAi pathway without sufficient evidence, as well as the claim regarding holocentromeric regions. We acknowledge these points and have taken them into consideration in our revisions.

While our investigation focused on an organism with holocentromeres and observed changes in holocentromeric protein localization, we recognize that further research is needed to determine whether the observed effect is specific to certain genomic sequences, say at the holocentromeric regions. In the revised manuscript, we have toned down the discussion to accurately reflect the findings and the need for additional investigation in this specific area.

Regarding the RNAi pathway, we have multiple lines of evidence that suggest the regulation occurs via CSR-1's RNAi pathway. We have reperformed the EGO-1 depletion experiment in Figure 6A using dsRNA injection, which consistently shows an increase in GFP::HCP-3 levels. EGO-1 is an upstream factor of the CSR-1 RNAi pathway, functioning as an RdRP that synthesizes siRNA that binds to CSR-1 to specify its target. Additionally, we have demonstrated that a CSR-1 mutant, which is deficient in cleaving RNA targets, is sufficient to trigger an increase in HCP-3 chromatin levels. Thus we believe CSR-1 represseses HCP-3

© 2024. Published by The Company of Biologists under the terms of the Creative Commons Attribution License (https://creativecommons.org/licenses/by/4.0/).

through its RNAi pathway.

Co-depletion of mRNAs of multiple genes by RNAi is not always comparable with single RNAi treatment. The authors should show the efficiency of the knockdown systematically, as for csr-1 and hrde-1 depletion in Figure S4B. In the hrde-1 example, the RNAi treatment was effective, but clearly less so in the co-depletion experiment than in the single depletion experiments. Without these controls, it is not possible to draw the conclusion from the experiments in Figure 5 that "CSR-1 promotes HCP-3 chromatin localization in embryos depleted of HCP-3 loading factors".

- We thank the reviewer for raising the importance of systematically comparing co-depletion experiments. As for *csr1 hrde-1*(RNAi), co-depletion led to a milder depletion when compared to the single knockdown, even though the dsRNA concentrations were made equal in the single and double RNAi. Although RT-qPCR reports the mRNA level after RNAi treatment, these mRNA level in the whole worm sample (including somatic tissues of injected worms) may not accurately reflect the knockdown efficiency in the embryos, where most of our assays are performed.

  To address the reviewer's core concern of proper comparison, and to ensure the functional impact of the double knockdown phenotype, we conducted a double knockdown control experiment where we co-deplete *knl-2* or *lin-53* with PRG-1, an argonuate which we found not affecting HCP- 3 levels (Figure 6C). Both co-depletion pairs (*knl-2 prg-1* and *knl-2 csr-1*) display kinetochore-null phenotypes. Importantly, the control pair (*knl-2 prg-1*) did not exhibit an increase in HCP-3 intensity, unlike the treatment pair (*knl-2 and csr-1*) as shown in Figure S5. This indicates that the increase of chromatin HCP-3 in *knl-2 csr-1* double knockdown was not caused by lower *knl-2* knockdown efficiency. The results from these control experiments support our conclusions drawn from the experiments in Figure 5.

In the stretched chromatin fiber experiment, all fibers, and not just one example, should be shown. Since the fibers likely represent different genomic regions, a direct comparison between wildtype control and csr-1 depletion is difficult. The increased number of foci is clearly visible, but it is impossible to conclude if this reflects an increase of HCP-3 at centromeric regions, or indeed a spread to additional genomic regions, as the authors propose.

- We agree that it will be helpful to include multiple fiber images to enable a better understanding of the overall trends and variations within our experimental data. In our revised manuscript, we have included multiple stretched chromatin fibers from both the wildtype control and csr-1 depletion conditions (Figure 3 and Figure S6).

  We also provide scatter plots in Figure S2C-D that plot H3 intensity and NND against H4 intensity, which indicates the difference in the degree of chromatin stretched, to show that even thinner chromatin (lower DAPI or H4 signal) have more HCP-3 foci in csr-1(RNAi). In addition, we now added a section in the Materials and Methods stating the criteria used to pick these regions for analysis. (line 499) "Our inclusion criteria for fiber: 1) Fibers with noticeable GFP::HCP-3, histone H4, and DAPI staining were imaged. 2) Fibers with similar chromatin width (determined from DAPI-staining) were selected and fiber areas were selected as 20-pixel-wide rectangles covering only the linear fibers for analysis."

  We thank the reviewer for pointing out that although the increased number of foci is evident, it is difficult to ascertain if this signifies an increase of HCP-3 specifically at centromeric region or a broader spread to additional genomic regions. We agree that it will be very useful to be able to pinpoint the holocentromere on the fiber. However, in C. elegans, performing centromere fluorescent in situ hybridization (FISH) with chromatin fiber is not straightforward, as C. elegans holocentromeres do not form on repeat sequences. One way to achieve this is by Oligopaint FISH of custom-defined centromere sequences [2, 3], and we are working on this in another project. We leave this out as the analysis of centromere sequence is out of the scope of this manuscript, and we have a separate ongoing project to address this. So here we pick fibers containing GFP foci to study the change in average distribution alongside the brighter HCP-3 signal on metaphase plate.

  We now added in discussion (line 293) "In the future, it will be important to determine the specific genomic sequences where HCP-3 binds when CSR-1 is depleted. This would help

© 2024. Published by The Company of Biologists under the terms of the Creative Commons Attribution License (https://creativecommons.org/licenses/by/4.0/).

distinguish between two potential scenarios: 1) the increase in HCP-3 foci occurs specifically within the centromeric regions, or 2) there is a broader redistribution and spread of HCP-3 binding to additional genomic regions beyond the centromeres. However, precisely pinpointing the locations of the holocentromeres on the chromatin fibers is technically challenging in C. elegans, as the holocentromeres do not form on typical repeat sequences. Future investigation will be needed to fully characterize the changes in HCP-3 localization under these conditions."

****

[Reviewer 3] Comments for the Author:
The manuscript's data are of high quality and are adequately controlled. Some comments on particular points follow that ought to be resolved before publication:

1- Several figures such as 2A show different images with different intensities, and conclusions are drawn based on the intensity being greater in one condition. For all such experiments, it should be stated explicitly whether the microscopy conditions were the same (for example, excitation light intensity and capture time, or antibody concentrations if appropriate) as they ought to be to interpret the results.

➢ We appreciate and agree with the reviewer's reminder regarding the need to explicitly state whether the microscopy conditions were the same for quantitative experiments. We agree that consistent microscopy conditions are crucial for proper interpretation of the results where signal intensities were compared.

We have now stated explicitly in the materials and methods section that the imaging settings, including excitation light intensity and capture time were kept the same for each protein and for comparisons between different treatments or strains. (Line 450) "All of our images were acquired using the same microscopy settings within an experiment set (excitation light power, exposure time, and number of z-stack, etc.) with its own untreated control."

We would like to highlight that in this revised manuscript, however, some of the newly added data (such as those shown in Figure 2A, Figure 5, and Figure 6) used a different microscope (due to unavailability of the previous one) thus resulting in different imaging conditions. To account for this variation, we have normalized the metaphase plate intensities to the average intensity of the untreated control samples taken using the same imaging conditions. This normalization allows us to calculate and display the metaphase plate intensity as a ratio or fold change relative to the untreated control.

We have included explicit statements in the Materials and Methods section (under "Image acquisition") to describe such differences in imaging conditions between the different figures and the normalization procedure employed for the metaphase plate intensities. (line 460) "In cases where data were taken using two different microscopes due to unavailability of the previous one, to account for this variation, we image also the control side by side for calculating the fold change of the intensity relative to its untreated control." This information will enhance the transparency and accuracy of the experimental design.

2- Some conclusions are drawn from double-RNAi experiments (Fig.1 C knl-1 csr-1(RNAi); however, since targeting two genes by RNAi often leads to sub-optimal knockdown, a test of RNAi effectiveness for lin-53 csr-1 and knl-2 csr-1 should be performed as it was for csr-1 hrde-1 (Figure S4).

➢ We thank reviewer for raising the importance of systematically comparing co-depletion experiments. We agree that the knockdown efficiency will be affected when targeting more than one gene by RNAi.

As for *csr1 hrde-1*(RNAi), co-depletion led to a milder depletion when compared to the single knockdown, even though the dsRNA concentrations were made equal. However, the RT-qPCR measures RNA level in the whole injected worms, including somatic tissues. Our phenotypic assays look at the HCP-3 localization inside the embryos produced from the injected worms, in which the knockdown is more thorough.

© 2024. Published by The Company of Biologists under the terms of the Creative Commons Attribution License (https://creativecommons.org/licenses/by/4.0/).

To determine if more HCP-3 are recruited to the kinetochore-null, over-compacted chromatin when CSR-1 is knocked down, we now included co-depletion control of another Argonaute PRG-1. In figure 6C, we have shown that PRG-1 does not affect chromatin HCP-3 level. Co-depletion of knl-2 and prg-1 shows kinetochore-null phenotype, while not having much HCP-3 on chromatin unlike the knl-2 and csr-1 co-depletion. Therefore, we are more confident to conclude that the increase in GFP::HCP-3 signal is a result of csr-1 depletion, but not an artifact manifested from a sub-optimal knockdown phenotype. We also did the control of lin-53 prg-1 co-depletion and see similar effects.

As for the double knockdown of *knl-1* and *csr-1* in Figure 1C, we have increased sample size and do not observe significant delay in csr-1 (RNAi). So we now reported this and moved the part to supplementary Figure S1E. However, this doesn't affect our main conclusion that CSR-1 is important for regulating chromatin HCP-3 level.

3- Fig. 2B : Please add detail in methods section explaining how line profiles were drawn and analyzed. In particular, how were the areas for line profiles selected (and how was bias in selection avoided)?

➢ We have now included the details of how chromosomal areas were selected for line profile analysis under Materials & Methods section "Profiling of prometaphase chromosome transverse intensity by linescan". The selection for line profiles requires chromosomes that appeared flat and have minimal rotation along the imaged x-y plane. We used only ROI from the same cell-stage between untreated and *csr-1*(RNAi). A 20-pixel-thick line was drawn across the selected chromosomes. A single line profile is the mean fluorescence intensity of the 20 pixels along the length of the drew line. As in *csr-1*(RNAi), the localization of the proteins remains bimodal, we combined multiple prometaphase chromosome line profiles for the same condition by setting the midpoint between the 2 peaks as the center and calculated the mean intensity at different distances away from the midpoint.

Additional minor comments:

1. Fig.1A, 3A, 4C - Please consider providing grayscale images instead of single-channel color images, for better contrast. Especially, the blue DAPI channel is very difficult to see.

➢ We have updated the panels to grayscale images for better contrast and visibility. Thank you for the suggestion.

2. 4C: Bar graphs at bottom can be made larger so labels are bigger/more legible.

➢ We have increased the font size and label size in the bar graphs at the bottom of Figure 4C to improve legibility.

3. Line 491: "(Spot Analysis)" - Please provide more detail about what kind of software was used for this analysis and how it was used.

➢ We apologize for the confusion caused by the previous description of "(Spot Analysis)" in line 491. It was misunderstood as the software used for downstream fiber GFP foci annotation and quantification. "(Spot Analysis)" is the name of our Nikon 80i Fluorescent Microscope's image acquisition software, not a software used for fiber analysis.

We have revised the Materials and methods section (Line 456): "For chromatin fibers assays, images were acquired with Nikon widefield fluorescence microscope (Nikon 80i Fluorescent Microscope) with 40x 0.75 NA air objective and the Nikon image acquisition software. The TIF files were then processed and quantified in the ImageJ platform." As for the procedure for fiber analysis using imageJ, it is under a separate section "Chromatin fiber preparation and analysis of the foci ROI" in the Materials and Methods.

4. The legends and figures for Supplemental Figures S2 and S3 are out of order.

➢ Thank you for pointing this out. We have fixed the page order for Figure S2 and S3 in the current version.

5. Figure 8: the legend states "Factors that we have tested to be not required or negative are

© 2024. Published by The Company of Biologists under the terms of the Creative Commons Attribution License (https://creativecommons.org/licenses/by/4.0/).

shown in red." but no such factors seem to be listed.

➢ We have removed the sentence stating "Factors that we have tested to be not required or negative are shown in red" from the figure legend for Figure 8 (which is Figure 7B now).

1. Singh, M., et al., *Translation and codon usage regulate Argonaute slicer activity to trigger small RNA biogenesis.* Nature Communications, 2021. **12**(1): p. 3492.
2. Gassmann, R., et al., *An inverse relationship to germline transcription defines centromeric chromatin in C. elegans.* Nature, 2012. **484**(7395): p. 534-7.
3. Steiner, F.A. and S. Henikoff, *Holocentromeres are dispersed point centromeres localized at transcription factor hotspots.* Elife, 2014. **3**: p. e02025.

Second decision letter

MS ID#: JOCES/2023/261895

MS TITLE: CSR-1 RNA interference pathway restricts holocentromere protein CENP-A/HCP-3 localization

AUTHORS: Charmaine Yan Yu Wong, Hok Ning Tsui, Yue Wang, and Karen Wing Yee Yuen
ARTICLE TYPE: Research Article

I am happy to tell you that your manuscript will be accepted for publication in Journal of Cell Science, pending standard publication integrity checks. Please pay attention to the drawing of the germ line in Figure 7B. We noticed that this is copied from Prosée et al., PLOS Biology, 2021, Figure 1.
https://doi.org/10.1371/journal.pbio.3000968. This publication is not referenced, neither for the figure, nor elsewhere in the manuscript. It should be, according to the copyright statement: Copyright: © 2021 Prosée et al. This is an open access article distributed under the terms of the Creative Commons Attribution License, which permits unrestricted use, distribution, and reproduction in any medium, provided the original author and source are credited.

Reviewer 1

Advance summary and potential significance to field

In this research article, Yan Yu Wong et al. analyse the role of the Argonaute protein CSR-1 in the localization of CENP-A/HCP-3 at holocentromeres in the C. elegans zygote. Employing a combination of genetic perturbations with microscopic observations, the authors first confirmed previously published findings on the role of CSR-1 in proper chromosome alignment and segregation in C. elegans zygotes. However, in contrast to previous publications, they attribute these defects to the ectopic over-recruitment of CENP-A/HCP-3, and downstream outer kinetochore components, at holocentromeres upon CSR-1 loss of function. By analyzing CENP-A/HCP-3 distribution on stretched chromatin fibers, the authors show that in absence of CSR-1, CENP-A/HCP-3 is primarily mislocalized at ectopic centromeric foci. The authors then show that this mislocalization is not associated to an effect of CSR-1 depletion on HCP-3 mRNA or protein levels, nor to a change in HCP-3 turnover at holocentromeres. Furthermore, HCP-3 over-recruitment could only partially be functionally linked to the CENP-A/HCP-3 loading factors LIN-53 and KNL-2. This last result implies the existence of a 'non-canonical' mechanism of CENP-A/HCP-3 loading at holocentromeres in C. elegans zygotes. Finally, Yan Yu Wong et al. show that the CENP-A/HCP-3 over-recruitment phenotype could be specifically attributed to the germline-expressed 'b' isoform of csr-1 (and not to the somatic 'a' isoform) and specifically to its 'slicing' activity, and that it could be recapitulated by depleting the RdRp EGO-1 suggesting that the associated RNAi pathway could be involved in the observed phenotype.

Comments for the author

The authors have addressed all my concerns. The manuscript is now suitable for publication.

© 2024. Published by The Company of Biologists under the terms of the Creative Commons Attribution License (https://creativecommons.org/licenses/by/4.0/).

Reviewer 2

Advance summary and potential significance to field

The summary of the advance made in this manuscript remains the same as in the first round of reviews.

Comments for the author

My major concerns about the fact that many of the manuscript‘s findings recapitulate previously published observations have not been addressed at all. It would have been interesting to hear from the authors where they see the advance in understanding the role of CSR-1 in holocentromere formation compared to the previous publications.

The remaining concerns have been addressed. Appropriate controls have been added (especially for the double RNAi experiments), the chromatin fiber analysis is much better documented, and the overall presentation of the findings is improved. I have no further comments.

Reviewer 3

Advance summary and potential significance to field

The argonaute protein CSR-1 is known to influence chromosome segregation, as loss of csr-1 gene function results in abnormal mitotic segregation, but the reasons for this have remained unclear. In this study evidence is provided that CSR-1 normally restricts the level of the centromeric histone HCP-3, as well as other kinetochore proteins, to chromosomes. The mechanism of CSR-1's activity remains unsolved, though the authors demonstrate that the RNA cleavage (slicer) activity is required, suggesting it may act through transcriptional regulation. This paper thus provides a potential link between chromosome segregation and a small RNA pathway (if it turns out that CSR-1's activity on chromosome segregation truly involves small RNAs).

Comments for the author

My comments in the earlier review, mainly concerning the microscopy conditions, selection of material for imaging and improvement of image presentation, and inquiries about confirming the effectiveness of two-target RNAi have been adequately addressed by addition or editing of the manuscript text.

NB: although the title in the Manuscript has been changed to "Argonaute CSR-1 restricts holocentromere protein CENP-A/HCP-3 localization", here in the Review system it's still shown as "CSR-1 RNA interference pathway restricts holocentromere protein CENP-A/HCP-3 localization", so please ensure the change is made.

© 2024. Published by The Company of Biologists under the terms of the Creative Commons Attribution License (https://creativecommons.org/licenses/by/4.0/).
